# TIPE1 inhibits osteosarcoma tumorigenesis and progression by regulating PRMT1 mediated STAT3 arginine methylation

**DOI:** 10.1038/s41419-022-05273-y

**Published:** 2022-09-23

**Authors:** Minghao Yang, Yuzhu Zhang, Guangping Liu, Ziqian Zhao, Jigang Li, Le Yang, Kui Liu, Wei Hu, Yunwei Lou, Jie Jiang, Qing Liu, Peiqing Zhao

**Affiliations:** 1 Department of Radiology, Yantai Affiliated Hospital of Binzhou Medical University, Yantai, 264100 PR China; 2 Center of Translational Medicine, Zibo Central Hospital Affiliated to Binzhou Medical University, Zibo, 255036 PR China; 3 The Second Medical College, Xinjiang Medical University, Urumqi, 830092 PR China; 4 Shandong First Medical University, Jinan, 250117 PR China; 5 School of Laboratory Medicine, Xinxiang Medical University, Xinxiang, 453003 PR China; 6 School of Chemistry and Chemical Engineering, Shandong University of Technology, Zibo, 255049 PR China

**Keywords:** Bone cancer, Methylation

## Abstract

Osteosarcoma (OS), the most common primary malignancy of the bone, has a poor prognosis due to its high mortality rate and high potential for metastasis. Thus, it is urgently necessary to explore functional molecular targets of therapeutic strategies for osteosarcoma. Here, we reported that TIPE1 expression was decreased in osteosarcoma tissues compared to normal and adjacent nontumor tissues, and its expression was negatively related to tumor stage and tumor size. Functional assays showed that TIPE1 inhibited osteosarcoma carcinogenesis and metastatic potential both in vivo and in vitro. Furthermore, we investigated that the STAT3 signaling pathway was significantly downregulated after TIPE1 overexpression. Mechanistically, TIPE1 bind to the catalytic domain of PRMT1, which deposits an asymmetric dimethylarginine (ADMA) mark on histone/non-histone proteins, and thus inhibited PRMT1 mediated STAT3 methylation at arginine (R) residue 688. This abolished modification decreased STAT3 transactivation and expression, by which subsequently suppressed osteosarcoma malignancy. Taken together, these data showed that TIPE1 inhibits the malignant transformation of osteosarcoma through PRMT1-mediated STAT3 arginine methylation and ultimately decreases the development and metastasis of osteosarcoma. TIPE1 might be a potential molecular therapeutic target and an early biomarker for osteosarcoma diagnosis.

## Introduction

Osteosarcoma (OS) is the most common primary malignancy of the bone and predominantly affects children and adolescents [1]. Approximately 15-20% of patients have metastases at the time of diagnosis, and in the past 30 years, the overall 5-year survival rate of patients with metastatic or relapsed osteosarcoma has been only 20% [2, 3]. Surgical resection and chemotherapy are still the primary strategies for osteosarcoma treatment. However, it is highly challenging to prevent osteosarcoma recurrence and treatment failure due to its invasive nature [4, 5]. Recently, some targeted agents, such as tyrosine kinase inhibitors (TKIs), mTOR inhibitors, and immune checkpoint inhibitors, have been explored in clinical trials [6–8]. Unfortunately, although some of these treatments may sustain disease control, most of them have failed to initiate objective responses [5]. Thus, it is urgent to explore the biological mechanisms underlying recurrent and refractory osteosarcoma, identifying novel potential therapeutic targets for osteosarcoma therapy.

The tumor necrosis factor alpha-induced protein 8 (TNFAIP8, TIPE) family is composed of four members, namely, tumor necrosis factor alpha-induced protein 8 (TIPE), tumor necrosis factor alpha-induced protein 8-like protein 1 (TIPE1), tumor necrosis factor alpha-induced protein 8-like protein 2 (TIPE2), and tumor necrosis factor alpha-induced protein 8-like protein 3 (TIPE3) [9, 10]. Although the numbers of this family have significant sequence homology, they are involved in different tumor biological activities [11]. Knockdown of TIPE1 expression can inhibit cell necroptosis and apoptosis [12], indicating that it might participate in the tumorigenic process. Previous study has demonstrated its role in tumorigenesis for the first time by showing that TIPE1 induces hepatocellular carcinoma cell apoptosis via the negative regulation of the Rac1 pathway [13]. Moreover, we showed that TIPE1 serves as an antioncogene in breast carcinoma [14, 15]. However, our group further revealed that TIPE1 can promote cervical cancer cell proliferation by suppressing p53 activity and increase nasopharyngeal carcinoma cell proliferation via the AMPK/mTOR signaling pathway [16**–**18]. Additionally, it has been reported that TIPE1 mRNA levels are increased in certain human tumor cells, including cervical cancer cells and tongue cancer cells [19], indirectly demonstrating that TIPE1 might serve as an oncogene in some types of tumors. Thus, in this context, we asked how TIPE1 participates in the development of osteosarcoma.

The signal transducer and activator of transcription (STAT) family of proteins was originally identified in the context of cytokine-mediated downstream signaling [20]. Specifically, STAT3 and STAT5, the two of the seven members of the STAT protein family, are the most important proteins for cancer progression [21, 22]. Specifically, the STAT3 pathway is a crucial regulator involved in osteosarcoma tumorigenesis [23, 24]. However, how STAT3 and its partners precisely regulate the occurrence and progression of osteosarcoma remains unclear. Here, we report that TIPE1 is a novel STAT3 pathway regulator and a tumor suppressor in osteosarcoma. Our data showed that TIPE1 inhibits osteosarcoma occurrence and metastasis both in vivo and in vitro. Mechanistically, TIPE1 inhibits the Protein arginine N-methyltransferase 1 (PRMT1) methyltransferase activity by binding to its catalytic domain, resulting abolished PRMT1-mediated STAT3 methylation at arginine (R) residue 688, and thus inhibition of STAT3 transactivation and expression. This study provides a proof of concept that targeting TIPE1 may be a therapeutic strategy for osteosarcoma.

## Results

### TIPE1 expression is decreased in osteosarcoma tissues and is negatively correlated with clinical stages

**Fig. 1:**
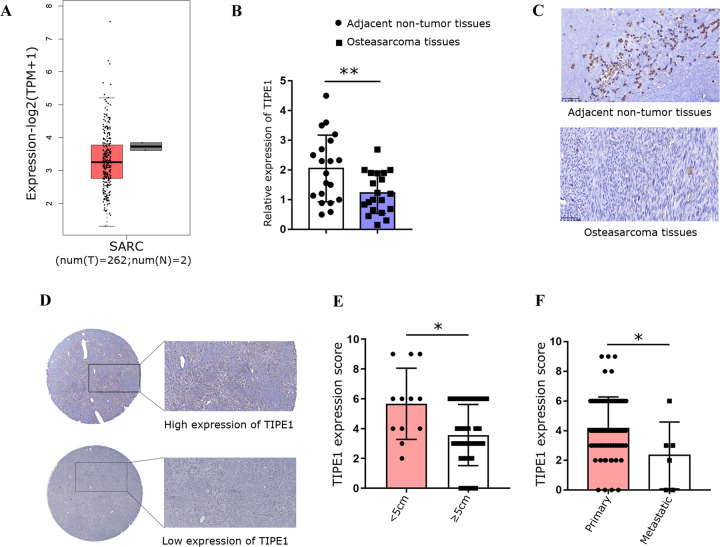
TIPE1 expression is down-regulated in osteosarcoma tissues and predicts a poor prognosis. **A** The level of TIPE1 expression in SARC tissues was lower than that in normal tissues in TCGA dataset. **B**, **C** The level of TIPE1 expression in osteosarcoma tissues was lower than that in adjacent nontumor tissues according to immunohistochemical staining. **D** The expression of TIPE1 in osteosarcoma tissue chips as shown by immunohistochemical staining. **E** Osteosarcoma patients with lower levels of TIPE1 were prone to developing large tumors by using immunohistochemical staining. **F** Osteosarcoma patients with lower levels of TIPE1 were more likely to have tumor metastasis by using immunohistochemical staining. **P < 0.01, *P < 0.05. The data represent the means ± SDs.

To date, there have been only a few articles about the role of TIPE1 in tumorigenesis, and its biological functions in cancers remain controversial. Therefore, we investigated how TIPE1 participates in the progression of osteosarcoma. First, we used the GEPIA website to determine the TIPE1 expression levels in sarcoma (SARC) tissues and normal tissues. The results showed that the levels of TIPE1 in sarcoma tissues were significantly lower than those in normal tissues (Fig. 1A). Furthermore, 20 osteosarcoma tissue samples and their corresponding nontumor tissue samples were collected to verify the above results. The results also showed that both the mRNA and protein expression levels of TIPE1 in osteosarcoma tissues were lower than those in adjacent nontumor tissues (Fig. 1B, C). Detailed analyses were conducted using a tissue microarray obtained from Zhongke Guanghua (Xi’an, China) Intelligent Biotechnology Co., Ltd. (detailed information is shown in Supplementary Table 1). Importantly, the TIPE1 levels were higher in the group with a tumor size <5 cm than in the group with a tumor size ≥5 cm (Fig. 1D, E). Furthermore, the protein expression of TIPE1 was significantly lower in metastatic osteosarcoma samples (clinical stage IV) than in primary osteosarcoma samples (clinical stage I and II) (Fig. 1F). Given that osteosarcoma in the late-stage present with stronger invasive capacity and metastatic potential than osteosarcoma in the earlier stage, TIPE1 might be involved in the progression and vascular invasion of osteosarcoma. However, due to the missing five-year survival data, we could not analyze the five-year survival of patients with osteosarcoma with respect to TIPE1 expression. To some extent, TIPE1 might play a critical role in the development and progression of osteosarcoma.

### TIPE1 exerts a negative effect on osteosarcoma cell tumorigenicity

**Fig. 2:**
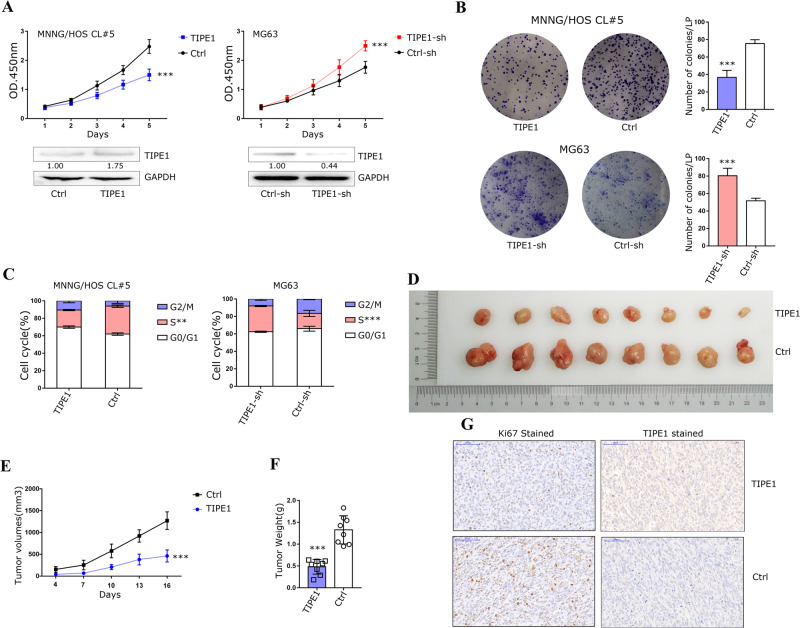
TIPE1 inhibits osteosarcoma cell proliferation. **A** Cell growth curves obtained using the CCK-8 method showed that TIPE1 decreased the proliferation rate of osteosarcoma cells compared to the control. **B** Clone formation assays showed that TIPE1 decreased the number of clones by osteosarcoma cells. **C** Cell cycle progression assay suggested that TIPE1 reduced the proportion of cells in the S phase, as shown by propidium iodide staining and flow cytometry. **D**–**F** TIPE1 overexpression reduced tumor growth compared to control (*n* = 8 mice/group), including tumor volumes and weight, in vivo. **G** TIPE1 overexpression downregulated the expression of Ki67 in xenograft tumor tissues, as determined by using immunohistochemical staining. ***P < 0.001, **P < 0.01. The data represent the means ± SDs of 3 replicates.

We further investigated endogenous TIPE1 expression levels in osteosarcoma cell lines by using western blotting and qPCR methods. As shown in Supplementary Figure 1, MNNG/HOS Cl #5 cell line presents a relative lower TIPE1 expression level compared with that MG63 cells have a higher TIPE1 expression level. Next, CCK-8, colony formation and cell cycle assays were performed to further substantiate the role of TIPE1 in osteosarcoma progression. The CCK-8 results showed that overexpression of TIPE1 decreased the proliferation rate of MNNG/HOS Cl #5 cells compared to the controls, indicating that TIPE1 inhibited the proliferation of osteosarcoma cells. Similarly, downregulation of TIPE1 expression in MG63 cells proved the above results (Fig. 2A). MNNG/HOS Cl #5 cells transfected with TIPE1 formed fewer colonies than the control cells, suggesting that TIPE1 could efficiently transform cells in the foci formation assay. On the other hand, the MG63 cells with knocked down TIPE1 expression formed more foci (Fig. 2B). Furthermore, cell cycle analysis showed that MNNG/HOS Cl #5 cells transfected with TIPE1 had a reduction in S phase, and downregulation of TIPE1 expression in MG63 cells significantly increased the proportion of S phase (Fig. 2C).

The results of an in vivo tumorigenesis experiment in nude mice showed that the nude mice injected with MNNG/HOS Cl #5 cells transfected with TIPE1 formed tumor with significantly smaller volumes and weights than the controls (Fig. 2D, E, and F). Ki67 staining of the tumor samples was performed to determine the cell proliferation rate, and the results indicated that TIPE1 might have tumor suppression potential in osteosarcoma (Fig. 2G). The above results suggested that TIPE1 significantly inhibits osteosarcoma cell proliferation both in vivo and in vitro.

### TIPE1 inhibits osteosarcoma cell migration, invasion and metastasis

**Fig. 3:**
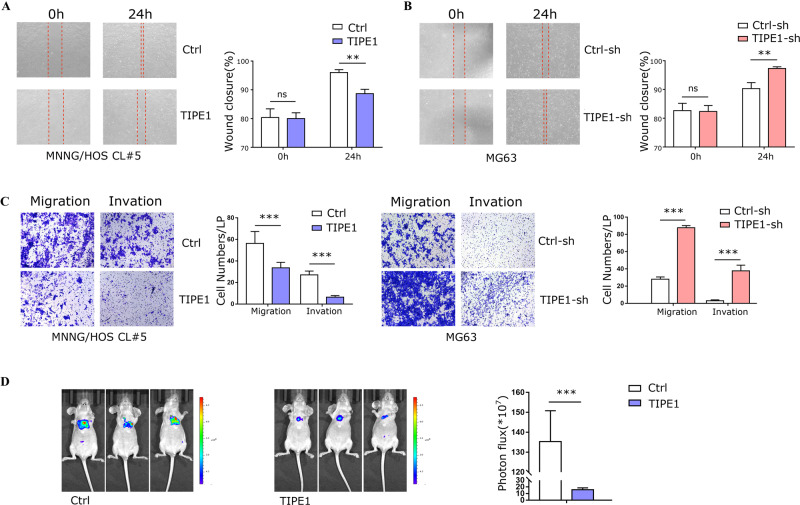
TIPE1 decreases osteosarcoma cell migration, invasion and metastasis. **A** Wound healing assay showed that overexpression of TIPE1 inhibited wound closure of MNNG/HOS Cl #5 cells. **B** Knocking down TIPE1 expression strongly improved wound closure in MG63 cells. **C** Transwell assays showed that cell migration and invasion were decreased in MNNG/HOS Cl #5 cells transfected with TIPE1, but these abilities were enhanced by knocking down TIPE1 expression in MG63 cells. **D** TIPE1 overexpression attenuated tumor metastasis in vivo as shown by bioluminescence images (*n* = 3 mice/group). ***P < 0.001, **P < 0.01. The data represent the means ± SDs of 3 replicates.

The expression of TIPE1 was significantly lower in metastatic osteosarcoma samples than in primary osteosarcoma samples. Thus, we further investigated the functions of TIPE1 in cell migration and invasion. As shown in Fig. 3A, overexpression of TIPE1 strongly inhibited wound closure in MNNG/HOS Cl #5 cells, while knockdown of TIPE1 expression in MG63 cells promoted wound closure (Fig. 3B). Transwell assays without Matrigel verified that TIPE1 had a negative effect on cell migration. Furthermore, Transwell chambers with Matrigel were used to confirm cell invasion. The results also showed that TIPE1 strikingly decreased cell invasion (Fig. 3C). Finally, we repeated the experiment in vivo using MNNG/HOS Cl #5 cells overexpressing TIPE1, and the results were similar to the in vitro studies (Fig. 3D). The above results suggested that TIPE1 decreases osteosarcoma cell metastatic potential, including the cell migration, invasion and metastasis abilities of osteosarcoma cells.

### TIPE1 inactivates the STAT3 signaling pathway

**Fig. 4:**
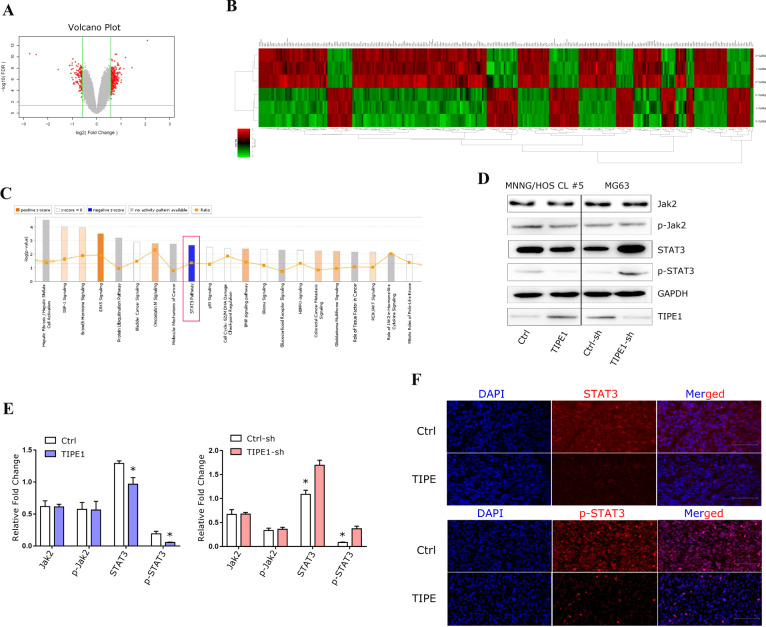
TIPE1 specifically inhibits STAT3 expression. Volcano plot **A**, Heatmap **B** and Ingenuity Pathway Analysis **C** showed that TIPE1 overexpression inhibited the STAT3 signaling pathway. **D**, **E** Western blotting assay showed that overexpression of TIPE1 specifically decreased total STAT3 and p-STAT3 levels in MNNG/HOS Cl #5 cells, while knockdown of TIPE1 expression increased total STAT3 and p-STAT3 expression in MG63 cells. **F** Immunofluorescence analysis suggested that overexpression of TIPE1 reduced the expression of total STAT3 and p-STAT3 in MNNG/HOS Cl #5 cells. The data represent the means ± SDs of 3 replicates.

The results from the in vitro and in vivo studies, together with the analysis of patient specimens, collectively showed that TIPE1 could suppress the development and progression of osteosarcoma. Thus, we performed gene expression and pathway analyses using Affymetrix GeneChip® Human Gene 2.0 ST arrays with MNNG/HOS Cl #5 cells transfected with the TIPE1 expression vector or control vector. We performed canonical pathway enrichment analysis in the ingenuity pathway analysis (IPA) of DEGs to determine the pathways most significantly affected by TIPE1. Among the top canonical pathways, the STAT3 pathway was inhibited (z-score of -0.816) after TIPE1 transfection (Fig. 4A, B and C). The expression levels of the Jak2/STAT3 pathway were further confirmed using Western blotting. The results showed that TIPE1 specifically decreased the expression level of total STAT3 and p-STAT3 in osteosarcoma cells, instead of the expression of total Jak2 and p-Jak2 (Fig. 4D, E). More interestingly, TIPE1 just suppressed the protein levels of STAT3, and its mRNA levels is not affected, indicating that TIPE1 might regulate STAT3 expression in a post-translational modification manner (Supplementary Figure 2). The immunofluorescence method also confirmed that TIPE1 down-regulated the expression level of total STAT3 and p-STAT3 in MNNG/HOS Cl #5 cells (Fig. 4F). STAT3 is both a proto-oncogene and molecular target of known oncogenes implicated in the development of osteosarcoma [25]. All of the above results indicated that TIPE1 could specifically inactivate the STAT3 protein and inhibit the malignant biological behavior of osteosarcoma.

### TIPE1 targets PRMT1 and suppresses osteosarcoma malignancy in a PRMT1-dependent manner

**Fig. 5:**
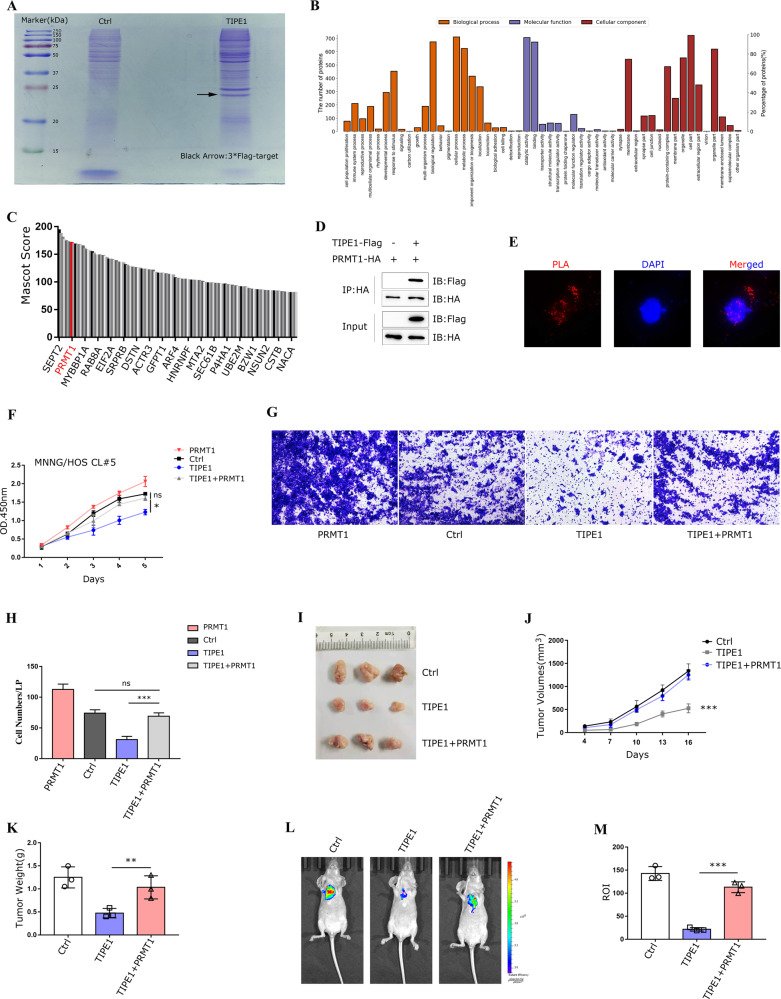
TIPE1 interacts with PRMT1 and suppresses osteosarcoma proliferation, invasion and metastasis in a PRMT1-dependent manner. **A** Immunopurification and mass spectrometry (MS) analysis of TIPE1-binding proteins. Cell extracts of MNNG/HOS Cl #5 cells ectopically expressing FLAG (Ctrl) or 3*FLAG/TIPE1 were purified with anti-FLAG M2 beads and eluted with FLAG peptide. The eluted proteins were resolved by SDS-PAGE and stained with Coomassie Brilliant Blue stained, and the protein bands were excised and analyzed by MS. **B**, **C** Gene Ontology functional annotation by using Blast2GO. **D**, **E** The interaction between PRMT1 and TIPE1 was confirmed by Co-IP and Duolink assays. **F** Overexpression of PRMT1 rescued the inhibitory activity of TIPE1 in MNNG/HOS Cl #5 cells according to CCK-8 assay. **G**, **H** PRMT1 overexpression restored the cell invasion that was inhibited by TIPE1. **I**, **J**, **K** PRMT1 overexpression reversed the TIPE1-mediated suppression of cell proliferation in vivo. **L**, **M** PRMT1 overexpression rescued the TIPE1-mediated suppression of cell metastasis in vivo (*n* = 3 mice/group). ***P < 0.001, **P < 0.01. The data represent the means ± SDs of 3 replicates.

In this case, we sought to determine the precise mechanisms by which TIPE1 decreased STAT3 signaling. Details, we conducted coimmunoprecipitation mass spectrometry (Co-IP/MS) analysis with the TIPE1-overexpressing MNNG/HOS Cl #5 cell line. Finally, the immunoprecipitates obtained from TIPE1 overexpression and control cells were separated using SDS-PAGE (Fig. 5A), and the target gel lanes were digested with trypsin for LC-MS/MS analyses. Interestingly, the Gene Ontology (GO) functional annotation using Blast2GO [26] showed that the proteins related to “Catalytic activity” and “binding” in the “Molecular function” module were increased, indicating that the function of proteins that interacted with TIPE1 might be related to catalytic activity (Fig. 5B). STAT3 is normally activated by interleukin family members and multiple pathways, such as pathways downstream of G‑protein-coupled receptors (GPCRs) and Toll-like receptors (TLRs), and its activation is characterized by tyrosine phosphorylation at residue Y705 together with Ser727 phosphorylation [20, 27]. In addition, STAT3 can be activated by methylation of its arginine residue(s) [28**–**30]. Thus, we selected the protein PRMT1 as a candidate TIPE1 interacting protein from a total of 729 peptides (Supplementary Table 2) primarily because STAT3 is a known substrate of PRMT1, and its activation is regulated by PRMT1 (Fig. 5C). In accordance, the Co-IP assays were conducted using HEK293 cells overexpressed with PRMT1-HA and TIPE1-Flag vectors, and the results revealed a specific interaction between them (Fig. 5D). Furthermore, we performed the Duolink in situ PLA to further study the interaction between TIPE1 and PRMT1 in the native cellular environment by using MNNG/HOS Cl #5 cells. Due to Duolink assay not only detects proteins interaction, but is visualized as individual fluorescent spots in cells, the results suggested that TIPE1 interacts with PRMT1 to form a red color complex both in the cytoplasm and nucleus (Fig. 5E).

Overexpression of PRMT1 has been demonstrated in many types of human cancers, and PRMT1 participates in certain cancers via its posttranslational modification functions [31, 32]. We thus postulated that TIPE1 inhibits the tumorigenesis and progression of osteosarcoma by regulating PRMT1-mediated STAT3 expression. To further verify this result, MNNG/HOS Cl #5 cells were transfected with TIPE1 with or without PRMT1. Consequently, by using CCK-8 method, we observed that cell proliferation was inhibited by TIPE1 but clearly increased by TIPE1-PRMT1 co-expression (cells transfected with PRMT1 alone was set as a positive control) (Fig. 5F), suggesting that TIPE1 suppresses osteosarcoma proliferation in a PRMT1-dependent manner. Similarly, PRMT1 significantly rescued the migration of MNNG/HOS Cl #5 cells, which had been suppressed by TIPE1 (Fig. 5G, H). Furthermore, we repeated the above experiments in vivo, and the results showed that both nude mouse tumor formation (Fig. 5I, J and K) and tumor metastasis (Fig. 5L, M) were restored by PRMT1 overexpression. The above results convincingly demonstrated that TIPE1 functions in a PRMT1-dependent manner.

### TIPE1 inhibits PRMT1 mediated STAT3 methylation at arginine 688

**Fig. 6:**
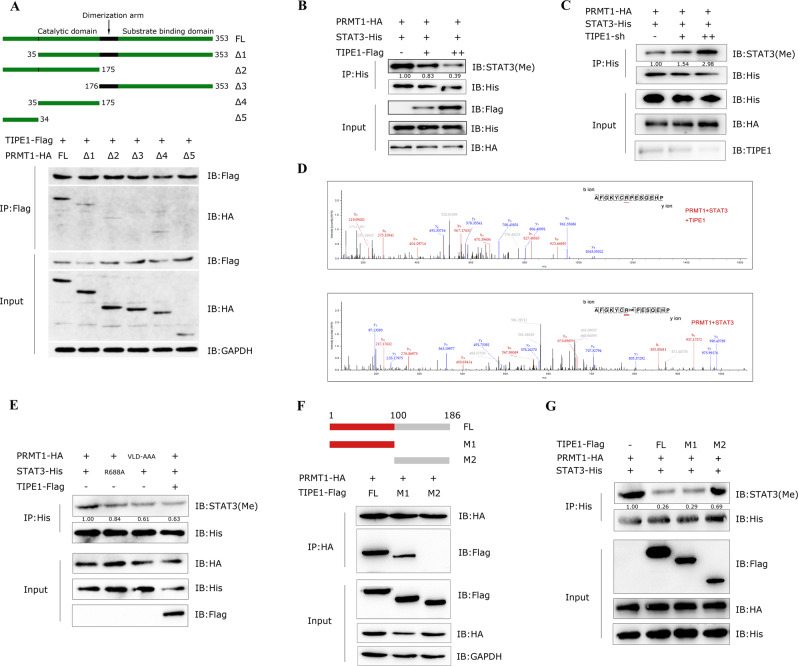
TIPE1 interacts with the catalytic domain of PRMT1 and inhibits its methyltransferase activity toward STAT3. **A** TIPE1 interacted with the catalytic domain of PRMT1 according to the Co-IP method. **B** IP and immunoblotting analysis of the levels of STAT3 methylation in HEK293T cells transfected with increasing doses of TIPE1. **C** IP and immunoblot analysis of the levels of STAT3 methylation in HEK293T cells transfected with increased doses of TIPE1-sh. **D** Mass spectrometry analysis of STAT3 peptide with (above) or without (below) TIPE1 plus PRMT1 enzyme showed that TIPE1 diminishes STAT3 arginine methylation at R688. **E** TIPE1, STAT3 R688A, and PRMT1(VLD-AAA) consistently reduces the STAT3 ADMA levels. **F** TIPE1 full length (FL) and M1 truncation, but not M2 interacted with PRMT1. **G** TIPE1 full length (FL) and M1 truncation, but not M2 downregulated STAT3 ADMA levels. The data represent the means ± SDs of 3 replicates.

Recent studies have highlighted that STAT3 functions as a transcription factor to activate target gene transcription and thus promotes tumor cell proliferation, survival, invasion, angiogenesis and immunosuppression. STAT3 is normally activated by interleukin family members and multiple pathways [20, 27]. In addition, STAT3 can be activated by methylation of its arginine residue(s) [28**–**30]. In this case, we sought to determine the precise mechanisms by which TIPE1 decreased STAT3 signaling. Protein arginine methylation is as a common posttranslational modification that regulates multiple cellular processes [33]. In this reaction, protein arginine methyltransferases (PRMTs) absolutely catalyze the transfer of a methyl group onto the arginine residues of histone or nonhistone proteins [34]. PRMT1 is the most predominant type I PRMT in mammalian cells, accounts for 85% of cellular PRMT activity [35, 36]. PRMT1 contains important domains, including the catalytic domain and the substrate-binding domain, and when its catalytic domain is bound, its methyltransferase activity can be inhibited. This repression results in functional changes in some PRMT1 substrates, especially STAT3 [29]. In the current case, we constructed a series of PRMT1 truncated vectors according to its functional domains to explore how TIPE1 affects the function of PRMT1. The results showed that TIPE1 can interact with truncated Δ1, Δ2 and Δ4 vectors of PRMT1, which both contains catalytic domain as shown in Fig. 6A, demonstrating that TIPE1 interacts with the catalytic domain of PRMT1 and might inhibit its methyltransferase activity. To further verify this hypothesis, we used the IP method to pull down total STAT3-His and detected its methylation levels using an antibody that specifically recognizes asymmetrically dimethylated arginine. As expected, the results showed that the levels of methylated STAT3 were decreased in a TIPE1 dose-dependent manner (Fig. 6B), which could be reversed by the transfection of TIPE1-sh into 293 T cells (Fig. 6C). Previous studies have proved that PRMT1 could promote the asymmetric arginine methylation of STAT3. However, the arginine methylated residues of STAT3 that modified by PRMT1 is not demonstrated [28, 30]. Next, STAT3 was pulled down from 293 T cells and subjected to LC-MS/MS analysis. We identified that arginine(R) residue at 688 of STAT3 was methylated after PRMT1 transfected, instead of dimethylated at arginine 688 when transfected combined with PRMT1 and TIPE1 (Fig. 6D). Previous research has shown that PRMT1 V84-L85-D86 to A-A-A mutation(VLD-AAA) renders it into a catalytically inactive form [36, 37]. To further validation, we transfected with VLD-AAA, mutative STAT3 R688A (R to Alanine [A]), and TIPE1 into 293 T cells. The results showed that both of those significantly reduced STAT3 ADMA levels (Fig. 6E), suggesting that R688 is the predominant ADMA residues for PRMT1 mediated STAT3 methylation, and TIPE1 can inhibit the arginine methylation of STAT3 by binding to the catalytic domain of PRMT1. Above results firmly demonstrated that TIPE1 has a characteristic to inhibit PRMT1 methyltransferase activity. Moreover, we verified whether TIPE1 competitively suppress the interaction between PRMT1 and STAT3. We transfected PRMT1-HA and STAT3-His together with increasing amount of TIPE1-Flag into HEK293T cells. The results showed that the interaction between PRMT1 and STAT3 was not enhanced or decreased regardless of the presence or absence of TIPE1(Supplementary Figure 3). Therefore, we believe that TIPE1 directly targets to the catalytic domain of PRMT1 to inhibit its methyltransferase activity.

Moreover, we constructed two truncated TIPE1 vectors, which named M1(1-100aa) and M2(101-186aa), respectively. The Co-IP results showed that PRMT1 interacted with full-length TIPE1(FL) and its M1 truncation, but not M2 (Fig. 6F). More interestingly, only full-length TIPE1 and its M1 truncation can abolish PRMT1 mediated STAT3 methylation (Fig. 6G). Above results, from the other hand, demonstrated that TIPE1, specifically its M1 truncation, abolished PRMT1 mediated STAT3 ADMA levels.

### TIPE1 decreases the activity and levels of STAT3

**Fig. 7:**
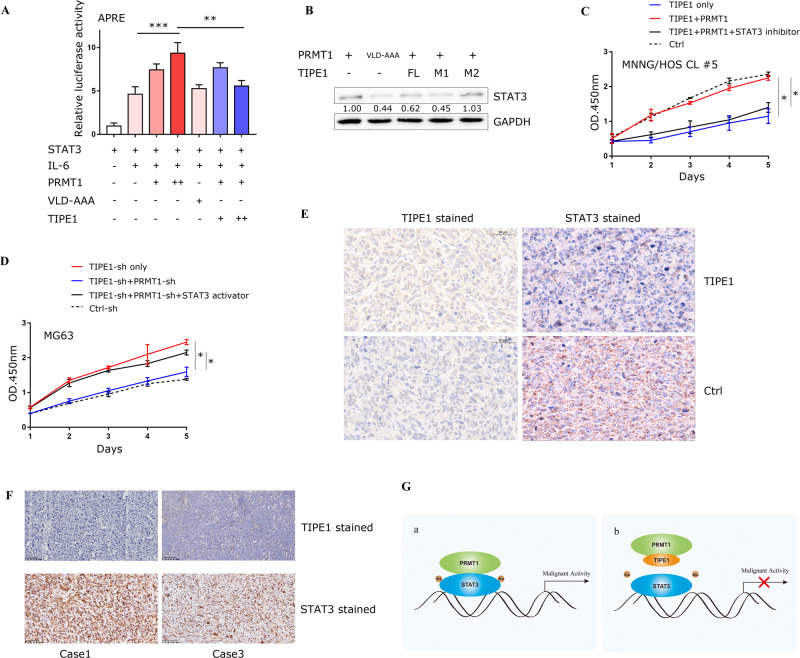
TIPE1 reduces STAT3 protein expression in osteosarcoma. **A** Effect of TIPE1 on the PRMT1-mediated transactivation of STAT3. Increasing amounts of PRMT1 together with STAT3 or TIPE1 were transfected into 293T cells. After transfection, the cells were treated with IL-6 (10 ng/ml) for 30 min. The luciferase activity of the APRE reporter gene was determined. PRMT1(VLD-AAA) as a negative control. **B** TIPE1 full length (FL) and M1 truncation, but not M2 reduced STAT3 protein expression by using Western blotting method. **C, D** STAT3 abrogates PRMT1 rescued the inhibition of osteosarcoma cell proliferation that caused by TIPE1 by using CCK8 method. (**E**) Analysis of tumor tissues from nude mice showed that the overexpression of TIPE1 decreased the expression of STAT3 as shown by immunohistochemical staining. **F** Analysis of 20 osteosarcoma examples showed that TIPE1 expression has a negative relationship with STAT3 expression, as assessed by immunohistochemistry assay. **G** Schematic diagram illustrating the mechanism by which TIPE1 inhibits osteosarcoma progression. The data represent the means ± SDs of 3 replicates.

To investigate the effect of TIPE1 on PRMT1 mediated the activation of STAT3, we transfected increasing amount of PRMT1-HA or TIPE1-Flag, together with STAT3 into 293 T cells, and the cells were treated with IL6 as previous demonstrated [29]. The luciferase activity of APRE reporter gene was determined to observe the effect of TIPE1 on PRMT1-induced transactivation of STAT3. The results showed that TIPE1 down-regulated PRMT1 induced STAT3 activity (Fig. 7A). Due to TIPE1 specifically decreased the protein levels of STAT3 as shown in Fig. 4D, we provided further evidences for this phenomenon by the western blot method in which the decreased expression of STAT3 that was inhibited by full-length TIPE1 or its M1 truncation, but not M2, with VLD-AAA served as a negative control (Fig. 7B). Moreover, we demonstrated that overexpression of PRMT1 can rescue TIPE1-caused the inhibition of cell proliferation. Interestingly, administration of STAT3 inhibitor surprisingly abolished this inhibition (Fig. 7C), and vice versa (Fig. 7D). Next, we stained the nude mouse tumors to analyze STAT3 expression, and the results showed that the groups overexpressing TIPE1 had lower STAT3 levels than the controls (Fig. 7E). We further detected the protein expression of TIPE1 and STAT3 in 20 osteosarcoma tissues by immunohistochemical staining. Interestingly, there was a significant negative correlation between the expression of TIPE1 and STAT3 (Fig. 7F). Finally, Therefore, we proved that TIPE1 downregulates STAT3 protein expression in clinical samples. Figure 7G schematic diagram illustrating how TIPE1 inhibits osteosarcoma progression.

## Discussion

In this study, we showed that TIPE1 inhibits STAT3 transactivation and expression via PRMT1, resulting in decreased osteosarcoma tumorigenesis and progression. To date, little information is available about the role of TIPE1. Its biological activity under both physiological and pathological conditions remains ambiguous [10]. A previous study suggested that TIPE1 suppresses osteosarcoma cancer cell growth by inhibiting MCP-1 expression in osteosarcoma cells, thus inhibiting the MCP-1/CCR2 axis via macrophage-osteosarcoma cell crosstalk [38]. However, it primarily showed that TIPE1 inhibits osteosarcoma progression by suppressing macrophage functions. How TIPE1 affects the tumorigenesis and progression of osteosarcoma cells has not been clearly demonstrated. More importantly, our Affymetrix Gene Chip results also demonstrated that the expression of MCP-1(CCL2) was not significantly changed after TIPE1 transfection, demonstrating that there might be other more important mechanisms involved in TIPE1-mediated osteosarcoma tumorigenesis and progression.

The JAK-STAT pathway was originally discovered in the context of IFN-α, IFN-γ and IL-6-mediated downstream signaling [20]. In addition to STAT3 playing a pivotal role during mammalian development, immune responses, inflammation and hematopoiesis, it has been implicated in the onset and progression of human cancers, including osteosarcoma [25, 39, 40]. However, because the diverse biology of STAT3 and its activators in cancer is complex, its biological functions in cancer cells are not clear. Traditionally, the JAK-STAT3 pathway is activated primarily by cytokines and growth factors, such as IL-6, Toll-like receptors (TLRs), and G-protein-coupled receptors (GPCRs) [41, 42]. In recent decades, reciprocal interactions between miRNAs and the JAK-STAT3 pathway have been identified, and these interactions play roles in regulating cancer-promoting inflammation and oncogenesis [20]. Some of the newly discovered upstream activators of JAK-STAT3 in cancer are crucial for designing novel and more effective cancer therapies. Thus, identifying and demonstrating novel regulators that contribute to JAK-STAT3 activation in cancer is urgent. In this study, we showed that TIPE1 could suppress the development and progression of osteosarcoma both in vivo and in vitro. Gene expression and Ingenuity Pathway Analysis (IPA) analyses demonstrated that the STAT3 pathway was inhibited after transfection of a TIPE1 expression vector, suggesting that TIPE1 could inactivate the STAT3 signaling pathway and inhibit the malignant biological behavior of osteosarcoma. However, the mechanism by which TIPE1 precisely regulates STAT3 has not been fully demonstrated.

Recent studies have revealed that posttranscriptional regulation plays a key role in STAT3 function. The N-methyladenosine (mA) demethylase FTO can regulate the JAK2-STAT3-C/EBPβ signaling axis, participating in the adipogenesis process in a posttranscriptional regulatory manner [43]. Acyl protein thioesterase 2 (APT2, also known as LYPLA2) depalmitoylates phosphorylated STAT3 (p-STAT3) and thus promotes Th17 cell differentiation [44]. More importantly, it has been reported that TR3 can bind to the catalytic domain of PRMT1 and repress PRMT1 methyltransferase activity. This process results in changes to PRMT1 substrates, including STAT3 [29]. Another article found that PRMT1 methylates the arginine residue(s) of STAT3 and thus increases its activity, resulting in the promotion of the differentiation of neural stem/precursor cells into astrocytes [30]. PRMT1 dysfunction is closely associated with various kinds of cancers [45]. For example, PRMT1-mediated FLT3 arginine methylation exacerbates acute lymphoblastic leukemia progression [32]. PRMT1 expression is increased in HCC samples and closely associated with clinical prognosis. PRMT1 might activate the STAT3 signaling pathway and participate in HCC progression [46]. Consequently, how PRMT1 affects HCC progression and metastasis by regulating STAT3 was not fully demonstrated in this study. Another report also showed that PRMT1 can methylate and activate STAT3 and contributes to osteosarcoma progression [28]. These results preliminarily suggested that PRMT1 regulates STAT3 by methylating its arginine residue(s) and thus participates in the progression of some diseases. However, which arginine residue(s) of STAT3 were modified by PRMT1 is not demonstrated [28, 30]. We further sought to determine the mechanisms by which TIPE1 decreases STAT3 signaling using Co-IP/MS analysis. Importantly, we found that “Catalytic activity” and “Binding” in the “Molecular function” module were increased after TIPE1 transfection, indicating that the function of proteins that interact with TIPE1 might be related to catalytic activity. Therefore, combined with previous studies, we believe that PRMT1 might be the most likely candidate protein that interacts with TIPE1 to participate in the procession of osteosarcoma. We further investigated how TIPE1 negatively regulates STAT3 via PRMT1. We transfected increasing levels of PRMT1 or TIPE1, together with STAT3, into 293 T cells and consequently administrated IL-6 as previously reported [29]. The results demonstrated that TIPE1 not only interacted with PRMT1 but also inhibits its activity, thus suppressing the transactivation of STAT3.

To determine whether TIPE1 inhibits osteosarcoma tumorigenesis and progression via PRMT1 specifically, we transfected TIPE1 into osteosarcoma cells with or without PRMT1. The results showed that cell proliferation was inhibited by TIPE1 but was rescued by co-transfection with TIPE1 and PRMT1. Similarly, PRMT1 significantly restored the osteosarcoma cell migration rate that was previously suppressed by TIPE1. The above results convincingly showed that TIPE1 functions in a PRMT1-dependent manner. Moreover, we constructed a series of truncated PRMT1 vectors to explore how TIPE1 affects the function of PRMT1. The results showed that TIPE1 can interact with the catalytic domain of PRMT1 and might inhibit its methyltransferase activity. Furthermore, we used the IP method to pull down total STAT3 and detected its methylation. As expected, the results showed that TIPE1 decreased the levels of methylated STAT3. More importantly, we identified that arginine(R) residue at 688 of STAT3 was methylated after PRMT1 transfected, and TIPE1 can abolish this methylation modification that caused by PRMT1. PRMT1 is the major methyltransferase that catalyzes both monomethylation and asymmetric dimethylation of arginine residues in eukaryotes. It typically functions as a regulator of chromatin dynamics via the methylation of histone H4 at arginine 3 (H4R3) [47]. To date, numerous nonhistone substrates, such as STAT3, have been identified [29, 30]. The variety of these substrates emphasizes the essential role of by PRMT1 in various cellular processes, such as transcriptional regulation [30, 48]. The canonical structure of PRMT1 contains three functional domains: the N-terminal methyltransferase domain constituting an AdoMet binding pocket; the C-terminal β-barrel domain that forms a cylindrical structure corresponding to the arginine-substrate binding site; and the α-helical dimerization arm that is responsible for PRMT1 dimerization [49]. In this study, we showed that TIPE1 interacted with the catalytic domain of PRMT1, which is located in its N-terminus, thus positively modulating its enzymatic activity toward a specific substrate, namely, STAT3. However, this pattern is different from that of histone H4; the latter could bind to the substrate-binding domain of PRMT1.This phenomenon explains why TIPE1 can inhibit the methyltransferase activity of PRMT1 via protein-protein interaction, rather than serving as a substrate. Moreover, how TIPE1 regulates PRMT1-medicated total STAT3 protein expression need to be further elucidated. Arginine methylation by PRMT1 participates in several cellular processes, including DNA damage repair, transcriptional regulation, apoptosis, and cell proliferation [50]. Previous study has revealed that PRMT1 regulates ATF4 protein stability through methylation of R239, and plays a protective role against ER stress-mediated cell death in the myocardium [51]. Thus, we speculated that TIPE1 downregulates the STAT3 protein levels, instead of mRNA levels, might via this manner as well. Therefore, we examined the relationship between TIPE1 and STAT3 expression, and the result also suggested a trend of a negative correlation between the expression of these molecules. However, this phenomenon needs to be further demonstrated in the future. More importantly, we demonstrated that overexpression of PRMT1 rescued TIPE1-caused the inhibition of cell proliferation, and administration of STAT3 inhibitor can abolish this inhibition, indicating that TIPE1 inhibits osteosarcoma proliferation primarily via PRMT1-mediated STAT3 suppression.

## Conclusions

We reported that TIPE1 inhibited cell proliferation, migration and invasion in the carcinogenesis of osteosarcoma by inhibiting PRMT1-mediated STAT3 function. Furthermore, we showed that low expression of TIPE1 was related to advanced-stages of osteosarcoma and that the expression of TIPE1 had a negative relationship with the expression of STAT3 in osteosarcoma patients. These results provide important insight into TIPE1-driven osteosarcoma pathogenesis and treatment and will identify potential therapeutic targets and diagnostic biomarkers of osteosarcoma.

## Materials and methods

### Cell culture, plasmid construction and transfection

Human osteosarcoma cells including MNNG/HOS Cl #5 and MG63 cells, and human embryonic kidney (HEK293T) cells were purchased from the Shanghai Institute of Cell Biology (Chinese Academy of Sciences, Shanghai, China). All the cells were cultured with DMEM supplemented with 10% FBS (Gibco) and 100 U/ml penicillin and 100 μg/ml streptomycin at 37 °C in a 5% CO_2_ atmosphere. The TIPE1 overexpression lentiviral vector (TIPE1), the TIPE1 expression knockdown vector (TIPE1-sh) and their control vectors were constructed by GeneChem company (Shanghai, China). TIPE1-Flag, STAT3-His, PRMT1-HA and their truncated vectors were constructed in our laboratory. After the cell lines were digested and seeded into plates, they were transfected with the indicated vectors according to the manufacturer’s protocol.

### Bioinformatics and microarray analysis

The GEPIA (Gene Expression Profiling Interactive Analysis, http://gepia.cancer-pku.cn/) website was used to evaluate the TIPE1 expression levels in sarcoma (SARC) and normal tissues. The relationship between TIPE1 and STAT3 expression levels was partly estimated using the ASSISTANT for Clinical Bioinformation website(https://www.aclbi.com/static/index.html#/), which is derived from The Cancer Genome Atlas (TCGA) dataset. MNNG/HOS Cl #5 cells were transfected with TIPE1 and control vectors for gene expression detection using the Affymetrix GeneChip® Human Gene 2.0 ST array, which was performed by the Genechem Company (Shanghai, China). Details, the detected differentially expressed transcripts were analyzed using Ingenuity Pathway Analysis (IPA) software. Enrichment of the focus genes in networks in IPA was assessed via Fisher’s exact test. To quantify the biological activity of pathways and main pathway regulators, the gene expression z-scores were calculated. Positive and negative z-scores indicate activation or inhibition of pathways, based on the relationships with differentially expressed genes (DEGs).

### Clinical samples

Twenty osteosarcoma tissue samples and their corresponding nontumor tissue samples were collected from Zibo Central Hospital affiliated to Binzhou Medical University for quantitative real-time PCR (qPCR) and Immunohistochemistry staining assay (IHC). Another seventy osteosarcoma samples, which included a total of 64 primary (clinical stage I and II) and 6 metastatic osteosarcomas (clinical stage IV) samples, were purchased from Zhongke Guanghua (Xi’an, China) Intelligent Biotechnology Co., Ltd. (#L714901) for IHC. The samples from Zibo Central Hospital were collected after obtaining informed consent, and their collection and use were approved by the Ethics Committee of Zibo Central Hospital. The study was also conducted in accordance with the Declaration of Helsinki.

### Real-time PCR

Total RNA was extracted from osteosarcoma tissues using TRIzol reagent (Invitrogen, USA), and then, cDNA was transcribed using the Prime Script RT Reagent Kit (TaKaRa, China). The cDNA was amplified with TIPE1 primers using Real SYBR Mixture (CoWin Bioscience, China) on an ABI PRISM 7500 instrument (Thermo Fisher, USA) to quantitate the relative mRNA expression of TIPE1 and STAT3. The Primer sequences were as follows: TIPE1: sense 5’-ACCTTCAGCACCAAGAGCCT-3’; antisense 5’-GACCAGGTTCTTGAGCATCT-3’. STAT3: sense 5’-GGGCCATCCTAAGCACAAAG-3’; antisense 5’-GGTCTTGCCACTGATGTCCTT-3’. The mRNA levels were normalized to the level of GAPDH, and the changes in expression were quantified using the 2^−∆∆Ct^ method.

### Immunohistochemical analysis

Immunohistochemistry (IHC) of osteosarcoma tissues was performed using anti-TIPE1 (1:500 dilution; sc-82761, Santa Cruz, USA), anti-STAT3(1:500 dilution; #9139, CST, USA), and anti-p-STAT3(1:500 dilution; #9145, CST, USA) antibodies. Immunohistochemistry staining was performed as we previously described [14]. Finally, each sample was analyzed with semiquantitative scoring criteria, in which both the intensity of the staining (0 = negative staining, 1= weak staining, 2 = moderate staining, and 3 = strong staining) and the percentage of positively stained cells (0 = less than 5% stained; 1 = 5 to 25% stained; 2 = 26 to 50% stained; 3 = 51 to 75% stained; and 4 = greater than 76% stained) were recorded. The staining index values (0-12) were obtained according to our previous report [16].

### Co-IP assay

HEK-293T cells were transfected with a TIPE1 plasmid containing a Flag tag and full-length PRMT1 or a series of truncated vectors containing an HA tag. Then, the cells were harvested at 24 h post-transfection. Cell lysates were incubated with a mouse anti-HA monoclonal antibody or rabbit anti-Flag polyclonal antibody for 2 h and subsequently incubated with protein G agarose beads (Roche, Switzerland) for 4 h. The protein-antibody complexes that were bound to the beads were eluted three times with cell lysis buffer and subjected to western blotting analysis to detect the relevant fusion proteins. The immunoreactive products were then detected by using an enhanced chemiluminescence kit (Thermo Fisher, USA).

### Cell proliferation, cell cycle and colony formation assay

Cell proliferation and viability were measured using the Cell Counting Kit-8 (Dojindo, Shanghai, China) method. For the colony formation assay, osteosarcoma cells (1 × 10^3^ cells per well) transfected with the TIPE1 overexpression vector (TIPE1) or interference vector (TIPE1-sh) were seeded in six-well plates and maintained in medium supplemented with 10% FBS for 7 days. In the results 7, STAT3 activator Colivelin (Selleck, USA, #867021-83-8) and inhibitor BP-1-102 (Selleck, USA, #1334493-07-0) was administrated as previous demonstrated. The colonies were then fixed using paraformaldehyde and stained with 0.1% crystal violet; the number of clones that contained more than 50 cells was counted and imaged. To analyze cell cycle progression, osteosarcoma cells were harvested, fixed and stained with propidium iodide (PI; Sigma, USA) according to the manufacturer’s instructions. Finally, the samples were evaluated with a flow cytometer (FACS Aria 2, BD, USA).

### Transwell assay

Tranwell with or without Matrigel (BD Biosciences, San Jose, CA, USA) were used to assess cell invasion and migration, respectively. Osteosarcoma cells in serum-free medium were seeded into the upper chambers (Corning, USA) with or without Matrigel at a density of 5 × 10^4^ cells per chamber. The lower chambers were then filled with medium supplemented with 10% FBS to serve as the chemoattractant. After incubation for 24 h, the nonmigrated or noninvaded osteosarcoma cells in the upper chambers were removed with cotton swabs, and migrated or invaded cells that had migrated through the membrane were fixed and stained with 0.1% crystal violet and observed using a microscope.

### Wound-healing assay

Cells were seeded in growth medium in 6-well plates. After confirming that the cells had grown and reached 90% confluence, the cells were then wounded by generating scratches with a sterile pipette tip. The wound area was observed at 0 h and 24 h and the migration rate was measured.

### Western blotting assay

Western blotting was conducted with the standard method. Ultimately, the PVDF membranes (Bio-Rad) were incubated with anti-STAT3 antibody (#9139, CST, USA), anti-p-STAT3(#9145, CST, USA), anti-Jak2 antibody (#3230, CST, USA), anti-p-Jak2 antibody (#3771, CST, USA), and tag antibodies, including an anti-HA antibody (Santa Cruz Biotechnology, USA), anti-Flag antibody (F7425, Sigma, USA) and anti-His antibody (Santa Cruz Biotechnology, USA). Asymmetric dimethyl-arginine (ASYM25, anti-Rme2,09-814) was purchased from Millipore (Billerica, MA). An anti-GAPDH antibody from Sigma was used as the internal control.

### Animal experiments

Osteosarcoma cells were subcutaneously injected into the dorsal flanks of four- to six-week-old male BALB/c nude mice to test the in vivo tumorigenicity of TIPE1 in osteosarcoma cell lines. The tumor volumes in all the groups were measured every 3 days. The mice were sacrificed and the tumor weights were measured when the tumor volumes reached ~80 mm^3^. For the pulmonary metastasis mouse model, an inoculum dose of 5 × 10^5^ MNNG/HOS Cl #5 osteosarcoma cells transfected with or without TIPE1 was intravenously injected through the tail vein of nude mice. Two weeks after the injection, the luminescence, which represents tumor metastases was quantified with the use of luciferin (12.5 mg by intraperitoneal injection). All the animal procedures were approved by the Animal Ethics Committee of Zibo Central Hospital, Binzhou Medical University. Nude mice were randomly allocated to experimental groups and no blinding method was used for injection. No statistical method was used to predetermine the sample size for the xenograft mice experiment, which was based on previous experimental observations. The sample size of each experiment is shown in the legend. There were no animal exclusion criteria.

### Co-immunoprecipitation mass spectrometry (Co-IP/MS) analysis of TIPE1-associated proteins

MNNG/HOS Cl #5 osteosarcoma cells were harvested 48 h after transfection with TIPE1-3 × FLAG lentivirus. The cells were washed twice with PBS and then digested and lysed in lysis buffer for 30 min at 4 °C. The lysed supernatant was incubated with FLAG-conjugated beads to acquire the coimmunoprecipitated products. A negative control was performed to collect the co-immunoprecipitated products without TIPE1-3 × FLAG. The beads were then washed 8 times using RIPA buffer and eluted with 3×FLAG peptide. The eluted proteins were resolved by SDS-PAGE and stained with Coomassie brilliant blue. Then, the bands were excised and digested with chymotrypsin and subjected to LC-MS/MS sequencing and data analysis by GeneChem (Shanghai, China). Functional annotation and classification of all the identified proteins were determined by using the Blast2GO program with the UniProt database. Pathway analyses were conducted using the search pathway tool of the KEGG mapper platform (http://www.genome.jp/kegg/mapper.html).

### Luciferase assay

Cells were transfected with the STAT3-His, TIPE1-Flag, PRMT1-HA vectors or the APRE luciferase reporter gene. Then, the cells were treated with IL-6 (10 ng/ml) for 30 min as previously reported [29]. The luciferase activity of the APRE reporter gene was determined to assess the transcriptional activity of STAT3.

### Duolink assay

Duolink in situ proximity ligation assays (PLAs) (DUO92008, Sigma-Aldrich) were performed with MNNG/HOS Cl #5 cells that overexpressed the TIPE1-Flag and PRMT1-HA proteins, as we previously reported [17].

### Statistical analysis

Data analysis was performed with GraphPad Prism (GraphPad Software, San Diego, CA). Chi-square tests were used to analyze the statistical correlation between the clinical parameters and the TIPE1 expression levels in tissue sections. Two-tailed Student’s t-tests were used to compare two groups. All the data are shown as the mean with standard deviations (SD) from at least three independent experiments. A *P* value of <0.05 was considered statistically significant for all tests.

## Supplementary information

Supplementary Materials

aj-checklist

Full Western Blots

## Data Availability

All data needed to evaluate the conclusions in the paper are present in the paper. Additional data related to this paper may be requested from the corresponding author.
