# Supplementary material for: TIPE1 inhibits osteosarcoma tumorigenesis and progression by regulating PRMT1 mediated STAT3 arginine methylation
Source: Cell Death Dis. 2022 Sep 23;13(9):815. doi: 10.1038/s41419-022-05273-y (PMC9508122; doi:10.1038/s41419-022-05273-y)
Supplement: Supplementary file 1 — Supplementary Materials [file 41419_2022_5273_MOESM1_ESM.docx]

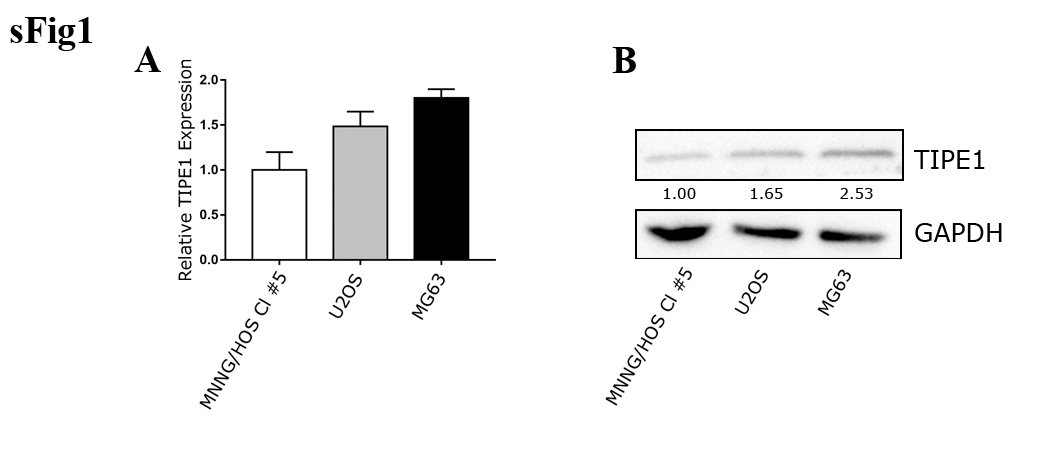


**Supplementary Figure 1. TIPE1 expression levels in osteosarcoma cell lines. (A)** TIPE1 mRNA levels in osteosarcoma cell lines determined by quantitative real-time PCR. **(B)** TIPE1 protein levels in osteosarcoma cell lines determined by Western blotting assays.


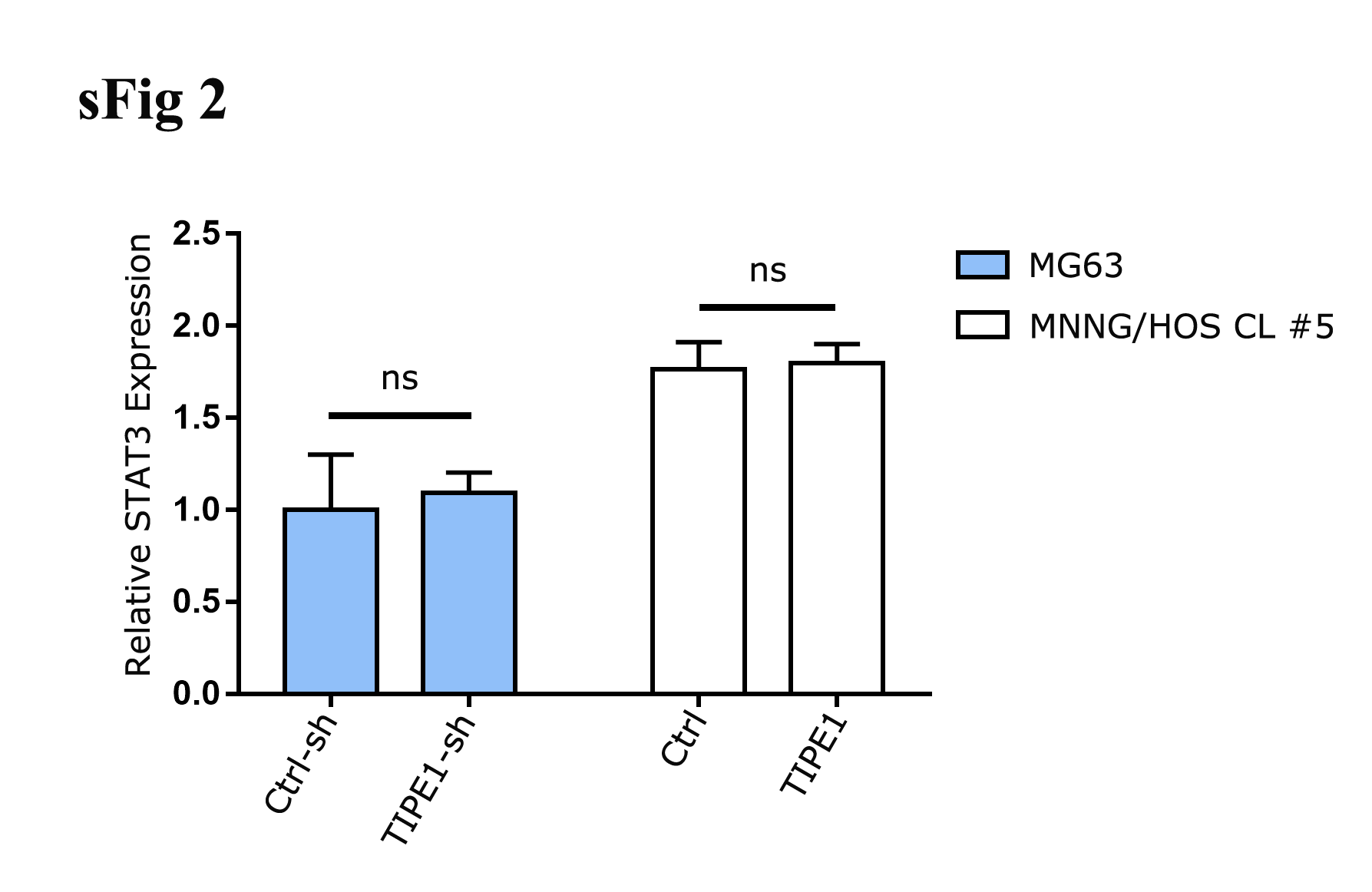


**Supplementary Figure 2.** The STAT3 mRNA levels after downregulation(left) or overexpression(right) of TIPE1. ns: no significance.


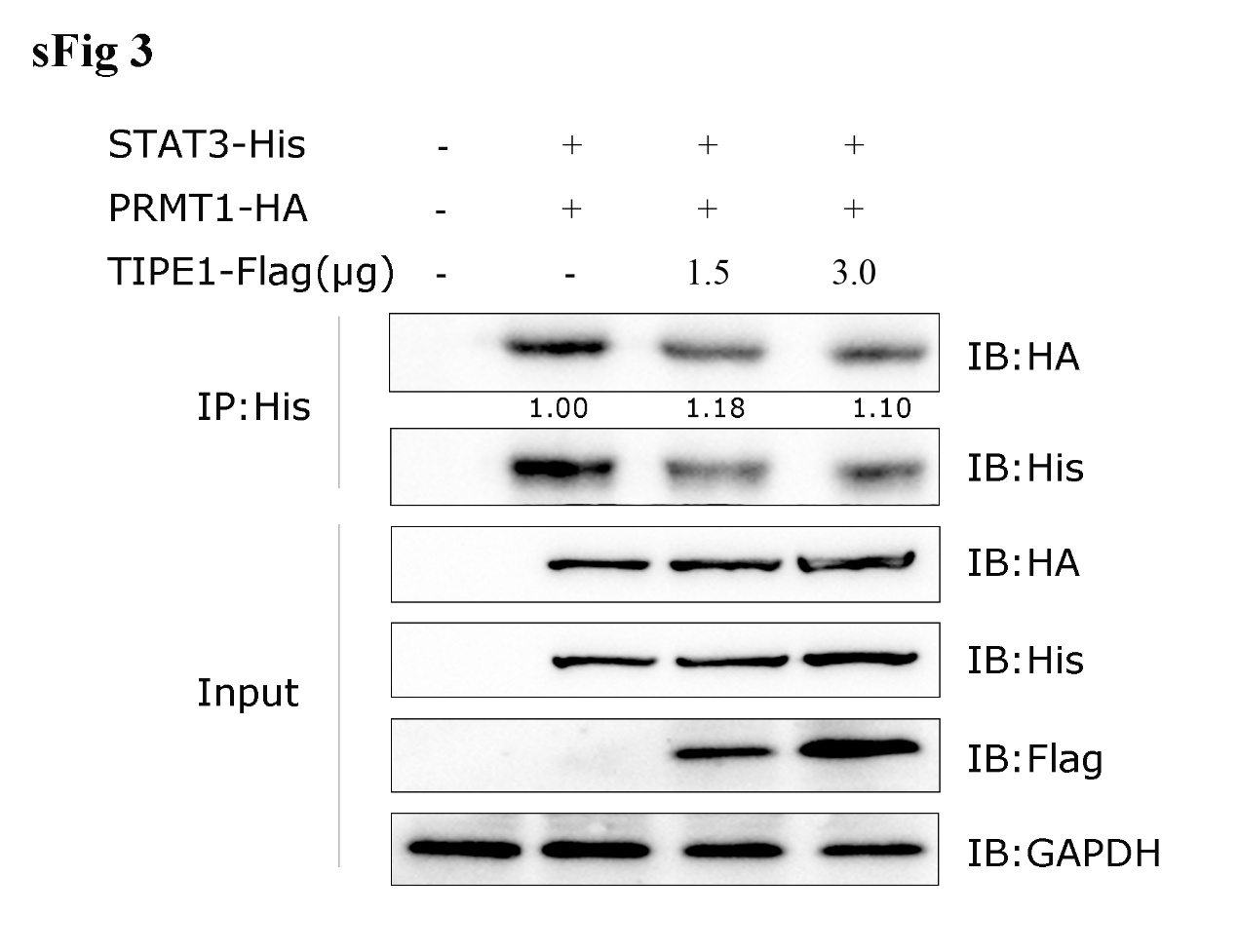


**Supplementary Figure 3.** IP and immunoblot analysis of the interaction between PRMT1 and STAT3 in HEK293T cells transfected with STAT3-His and PRMT1-HA in the presence of increasing amount of TIPE1-Flag.

**Supplementary Table 1: Characteristics of patients with osteosarcoma in tissue microarray**

|  | **Age(years)** | **Gender** | **Location** | **TNM stage** | **Clinical stage** | **Lymph node metastasis** | **Tumor size (cm)** |
| --- | --- | --- | --- | --- | --- | --- | --- |
| A1 | 41 | Male | Left femur | T1N0M0 G2 | IA | None | 4.0 |
| A2 | 59 | Female | Right femur | T1N0M0 G2 | IA | None | 12.0 |
| A3 | 50 | Female | Right femur | T1N0M0 G3 | IIA | None | 2.0 |
| A4 | 17 | Male | Left tibia | T1N0M0 G3 | IIA | None | 4.5 |
| A5 | 47 | Male | Left femur | T1N0M0 G3 | IIA | None | 8.0 |
| A6 | 35 | Male | Left chest wall | T1N0M0 G3 | IIA | None | 8.0 |
| A7 | 12 | Female | Femur | T1N0M0 G3 | IIA | None | 7.0 |
| A8 | 34 | Female | Right femur | T1N0M0 G3 | IIA | None | *(Data missing) |
| A9 | 18 | Female | Left femur | T1N0M0 G3 | IIA | None | 2.0 |
| B1 | 57 | Male | Femur | T1N0M0 G3 | IIA | None | 6.0 |
| B2 | 18 | Male | Femur | T1N0M0 G3 | IIA | None | 6.0 |
| B3 | 15 | Male | Humerus | T1N0M0 G3 | IIA | None | *(Data missing) |
| B4 | 56 | Female | Left ilium | T1N0M0 G3 | IIA | None | 6.0 |
| B5 | 52 | Male | Scapula | T1N0M0 G3 | IIA | None | 8.0 |
| B6 | 10 | Female | Right tibia | T1N0M0 G3 | IIA | None | 4.0 |
| B7 | 69 | Male | Right femur | T1N0M0 G3 | IIA | None | 4.0 |
| B8 | 17 | Male | Left femur | T1N0M0 G3 | IIA | None | 8.0 |
| B9 | 14 | Female | Right femur | T1N0M0 G3 | IIA | None | 5.0 |
| C1 | 15 | Male | Right femur | T1N0M0 G3 | IIA | None | 6.0 |
| C2 | 31 | Female | Left femur | T1N0M0 G3 | IIA | None | 4.6 |
| C3 | 64 | Male | Right lower extremity | T1N0M0 G3 | IIA | None | 7.0 |
| C4 | 24 | Male | Right femur | T1N0M0 G3 | IIA | None | 8.0 |
| C5 | 32 | Male | Left sixth frame | T1N0M0 G3 | IIA | None | 4.0 |
| C6 | 30 | Male | Right tibia | T1N0M0 G3 | IIA | None | 5.0 |
| C7 | 14 | Female | Left tibia | T1N0M0 G3 | IIA | None | 3.0 |
| C8 | 62 | Female | Left femur | T1N0M0 G3 | IIA | None | 4.0 |
| C9 | 13 | Female | Left femur | T1N0M0 G3 | IIA | None | 9.0 |
| D1 | 15 | Female | Left femur | T1N0M0 G3 | IIA | None | *(Data missing) |
| D2 | 31 | Male | Left humerus | T1N0M0 G3 | IIA | None | 6.0 |
| D3 | 38 | Female | Right scapula | T1N0M0 G3 | IIA | None | 7.0 |
| D4 | 64 | Male | Left femur | T1N0M0 G3 | IIA | None | 6.0 |
| D5 | 46 | Male | Right femur | T1N0M0 G3 | IIA | None | *(Data missing) |
| D6 | 33 | Male | Upper jaw | T1N0M0 G3 | IIA | None | *(Data missing) |
| D7 | 42 | Male | Left femur | T2N0M0 G3 | IIB | None | 6.0 |
| D8 | 14 | Female | Left tibia | T2N0M0 G3 | IIB | None | *(Data missing) |
| D9 | 51 | Male | Right fibula | T2N0M0 G3 | IIB | None | *(Data missing) |
| E1 | 32 | Female | Left femur | T2N0M0 G3 | IIB | None | *(Data missing) |
| E2 | 35 | Female | Thoracic vertebra | T2N0M0 G3 | IIB | None | 7.0 |
| E3 | 19 | Male | Left femur | T2N0M0 G3 | IIB | None | 20.0 |
| E4 | 28 | Male | Right lower extremity | T2N0M0 G3 | IIB | None | 6.0 |
| E5 | 36 | Male | Left femur | T2N0M0 G3 | IIB | None | 7.0 |
| E6 | 28 | Male | Left humerus | T2N0M0 G3 | IIB | None | 3.0 |
| E7 | 29 | Male | Femur | T2N0M0 G3 | IIB | None | 6.0 |
| E8 | 42 | Male | Left femur | T2N0M0 G3 | IIB | None | 5.5 |
| E9 | 19 | Male | Left tibia | T2N0M0 G3 | IIB | None | *(Data missing) |
| F1 | 60 | Male | Right tibia | T2N0M0 G3 | IIB | None | 6.0 |
| F2 | 28 | Female | Left humerus | T2N0M0 G3 | IIB | None | 6.0 |
| F3 | 10 | Male | Left tibia | T2N0M0 G3 | IIB | None | 6.0 |
| F4 | 19 | Male | Left fibula | T2N0M0 G3 | IIB | None | *(Data missing) |
| F5 | 16 | Male | Left femur | T2N0M0 G3 | IIB | None | *(Data missing) |
| F6 | 21 | Male | Left femur | T2N0M0 G3 | IIB | None | *(Data missing) |
| F7 | 18 | Male | Right humerus | T2N0M0 G3 | IIB | None | *(Data missing) |
| F8 | 19 | Male | Left fibula | T2N0M0 G3 | IIB | None | *(Data missing) |
| F9 | 16 | Female | Left femur | T2N0M0 G3 | IIB | None | *(Data missing) |
| G1 | 27 | Male | Right humerus | T2N0M0 G3 | IIB | None | 10.0 |
| G2 | 17 | Male | Left femur | T2N0M0 G3 | IIB | None | *(Data missing) |
| G3 | 37 | Female | Right femur | T2N0M0 G3 | IIB | None | 9.0 |
| G4 | 12 | Male | Left femur | T2N0M0 G3 | IIB | None | 10.0 |
| G5 | 32 | Female | Right humerus | T2N0M0 G3 | IIB | None | *(Data missing) |
| G6 | 20 | Female | Right tibia | T2N0M0 G3 | IIB | None | *(Data missing) |
| G7 | 16 | Male | Right femur | T2N0M0 G3 | IIB | None | *(Data missing) |
| G8 | 15 | Female | Left knee | T2N0M0 G3 | IIB | None | *(Data missing) |
| G9 | 12 | Male | Left femur | T2N0M0 G3 | IIB | None | 10.0 |
| H1 | 15 | Female | Left knee | T2N0M0 G3 | IIB | None | *(Data missing) |
| H2 | 16 | Female | Left femur | T3N1M0 G3 | IVB | Yes | 4.0 |
| H3 | 22 | Male | Right ulna | T3N1M0 G3 | IVB | Yes | *(Data missing) |
| H4 | 15 | Male | Lower jawbone | T2N1M0 G3 | IVB | Yes | *(Data missing) |
| H5 | 17 | Male | Left humerus | T3N1M0 G3 | IVB | Yes | 5.0 |
| H6 | 52 | Male | Left third frame | T3N1M0 G3 | IVB | Yes | 6.0 |
| H7 | 28 | Male | Right lower extremity | T3N1M0 G3 | IVB | Yes | 6.0 |
| H8 | 40 | Male | Normal bone tissue | - | - | - | - |

**Supplementary Table 2: Candidate interacting proteins of TIPE1 identified by mass spectrometry**

| **Accession** | **Protein name** | **Gene name** | **MW(KDa)** | **Sequence coverage(%)** |
| --- | --- | --- | --- | --- |
| P07437 | Tubulin beta chain | TUBB | 49.639 | 36.036036 |
| Q9BVA1 | Tubulin beta-2B chain | TUBB2B | 49.921 | 27.865169 |
| Q13885 | Tubulin beta-2A chain | TUBB2A | 49.875 | 27.865169 |
| Q13509 | Tubulin beta-3 chain | TUBB3 | 50.4 | 27.111111 |
| Q8WVP5 | Tumor necrosis factor alpha-induced protein 8-like protein 1 | TNFAIP8L1 | 20.814 | 29.569892 |
| P0CG39 | POTE ankyrin domain family member J | POTEJ | 117.315 | 5.5876686 |
| Q9BUF5 | Tubulin beta-6 chain | TUBB6 | 49.825 | 18.38565 |
| P68366 | Tubulin alpha-4A chain | TUBA4A | 49.892 | 25.669643 |
| P06899 | Histone H2B type 1-J | HIST1H2BJ | 13.896 | 41.269841 |
| Q16658 | Fascin | FSCN1 | 54.496 | 19.066937 |
| P16403 | Histone H1.2 | HIST1H1C | 21.352 | 25.352113 |
| A0A075B6S2 | Immunoglobulin kappa variable 2D-29 | IGKV2D-29 | 13.135 | 16.666667 |
| P62753 | 40S ribosomal protein S6 | RPS6 | 28.663 | 25.301205 |
| Q12905 | Interleukin enhancer-binding factor 2 | ILF2 | 43.035 | 17.435897 |
| Q13310 | Polyadenylate-binding protein 4 | PABPC4 | 70.738 | 11.335404 |
| P07951 | Tropomyosin beta chain | TPM2 | 32.831 | 15.492958 |
| Q07065 | Cytoskeleton-associated protein 4 | CKAP4 | 65.983 | 15.282392 |
| P30566 | Adenylosuccinate lyase | ADSL | 54.854 | 9.0909091 |
| P23381 | Tryptophan--tRNA ligase, cytoplasmic | WARS | 53.132 | 16.985138 |
| Q15019 | Septin-2 OS=Homo sapiens | SEPT2 | 41.461 | 14.68144 |
| Q9NTJ3 | Structural maintenance of chromosomes protein 4 | SMC4 | 147.091 | 5.6677019 |
| Q9P2J5 | Leucine--tRNA ligase, cytoplasmic | LARS | 134.379 | 6.037415 |
| Q14839 | Chromodomain-helicase-DNA-binding protein 4 | CHD4 | 217.867 | 2.8242678 |
| P08195 | 4F2 cell-surface antigen heavy chain | SLC3A2 | 67.952 | 15.079365 |
| O15067 | Phosphoribosylformylglycinamidine synthase | PFAS | 144.643 | 4.5590433 |
| Q92598 | Heat shock protein 105 kDa | HSPH1 | 96.804 | 11.305361 |
| Q99873 | Protein arginine N-methyltransferase 1 | PRMT1 | 42.434 | 23.450135 |
| Q13263 | Transcription intermediary factor 1-beta | TRIM28 | 88.493 | 12.335329 |
| O60701 | UDP-glucose 6-dehydrogenase | UGDH | 54.989 | 11.336032 |
| Q02952 | A-kinase anchor protein 12 | AKAP12 | 191.367 | 5.7239057 |
