# Supplementary figures and images for: TIPE1 inhibits osteosarcoma tumorigenesis and progression by regulating PRMT1 mediated STAT3 arginine methylation
Source: Cell Death Dis. 2022 Sep 23;13(9):815. doi: 10.1038/s41419-022-05273-y (PMC9508122; doi:10.1038/s41419-022-05273-y)

**Fig. 2**

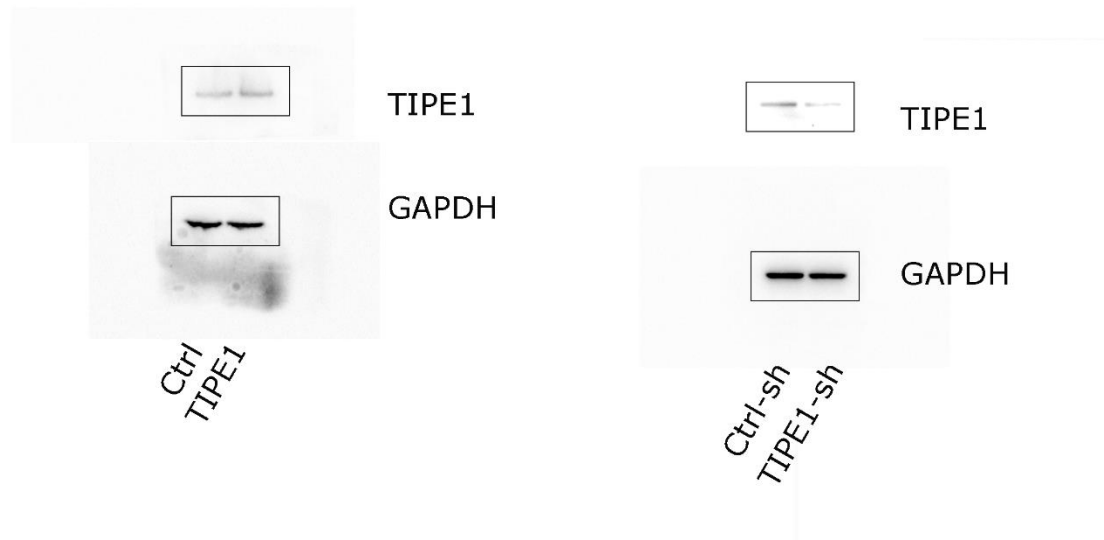

**Fig. 4**

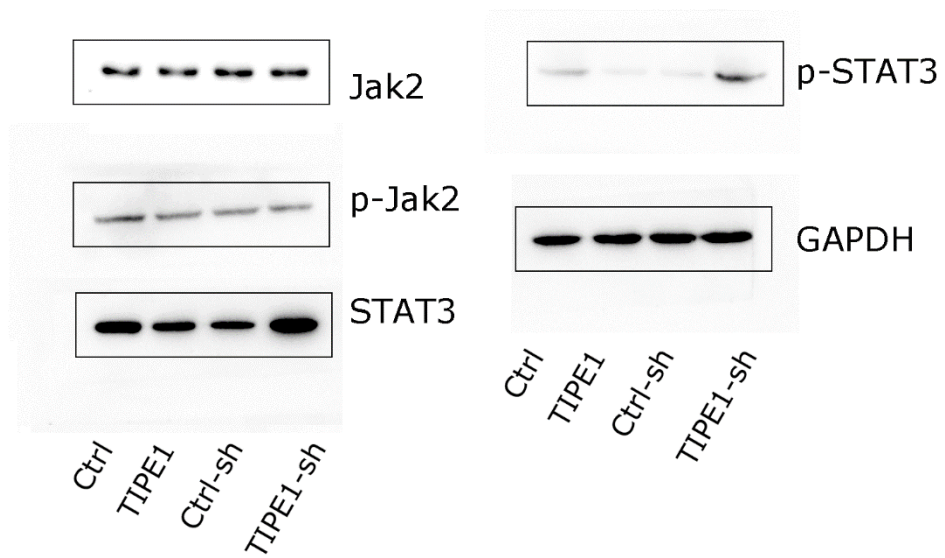

**Fig. 5**

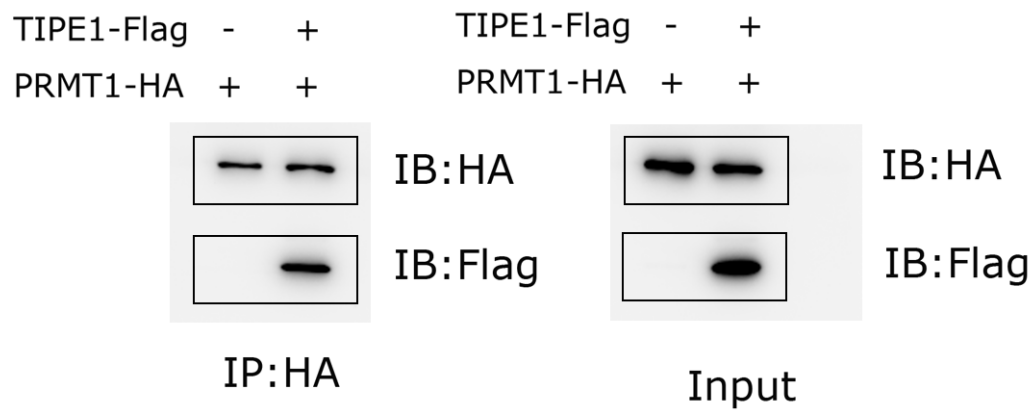

**Fig. 6A**

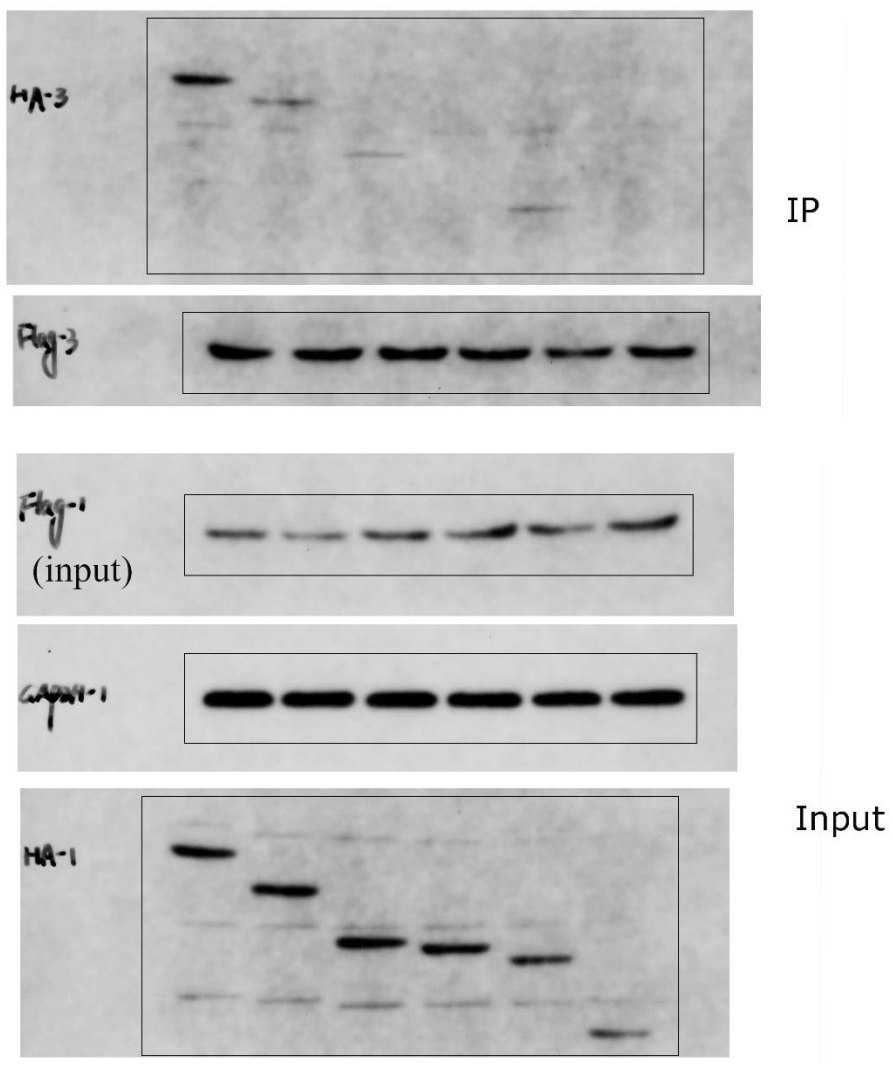

**Fig. 6B**

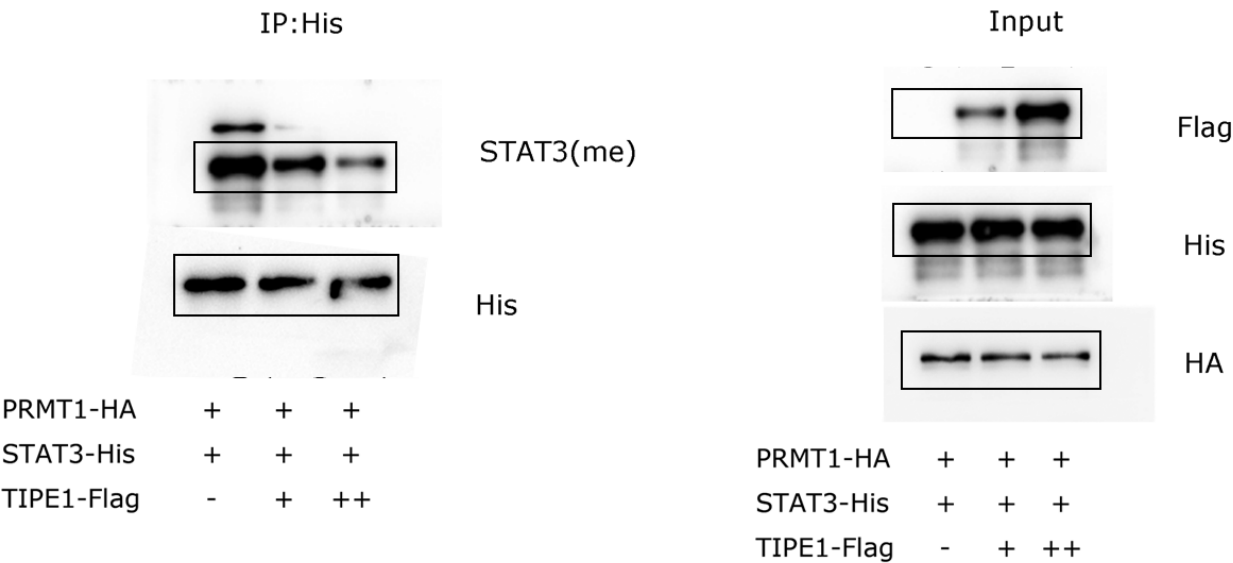

**Fig. 6C**

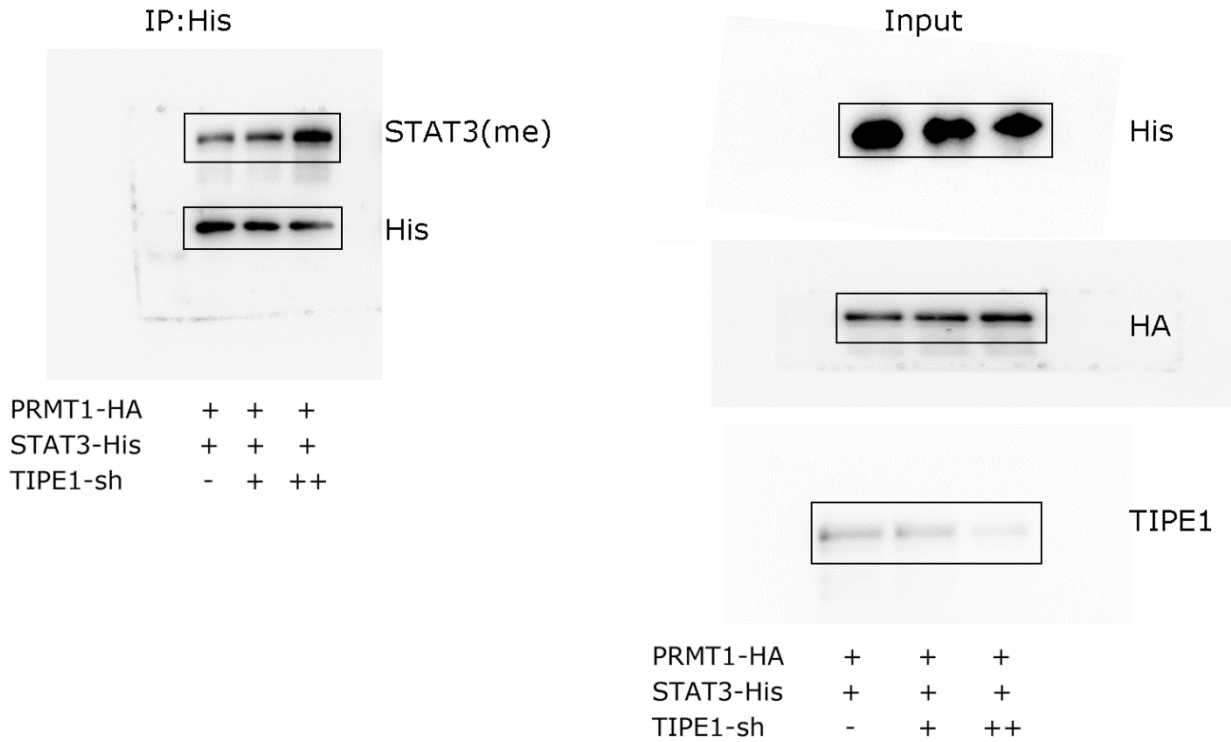

**Fig. 6E**

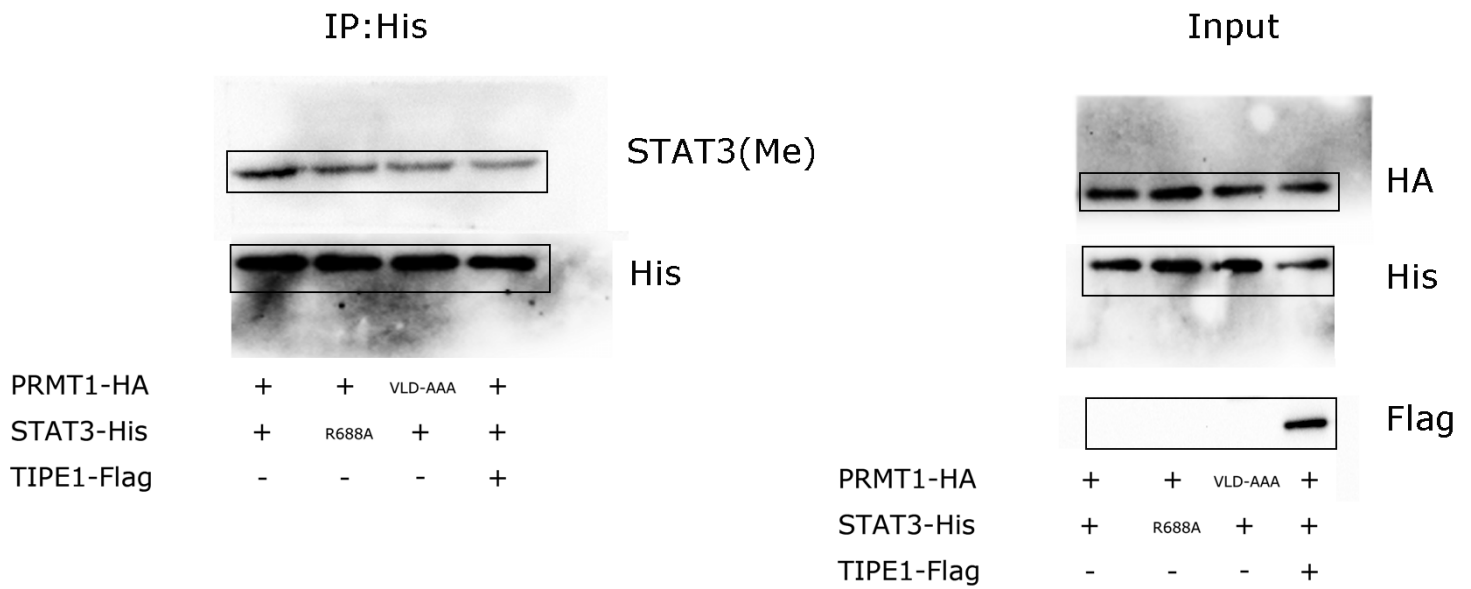

**Fig. 6F**

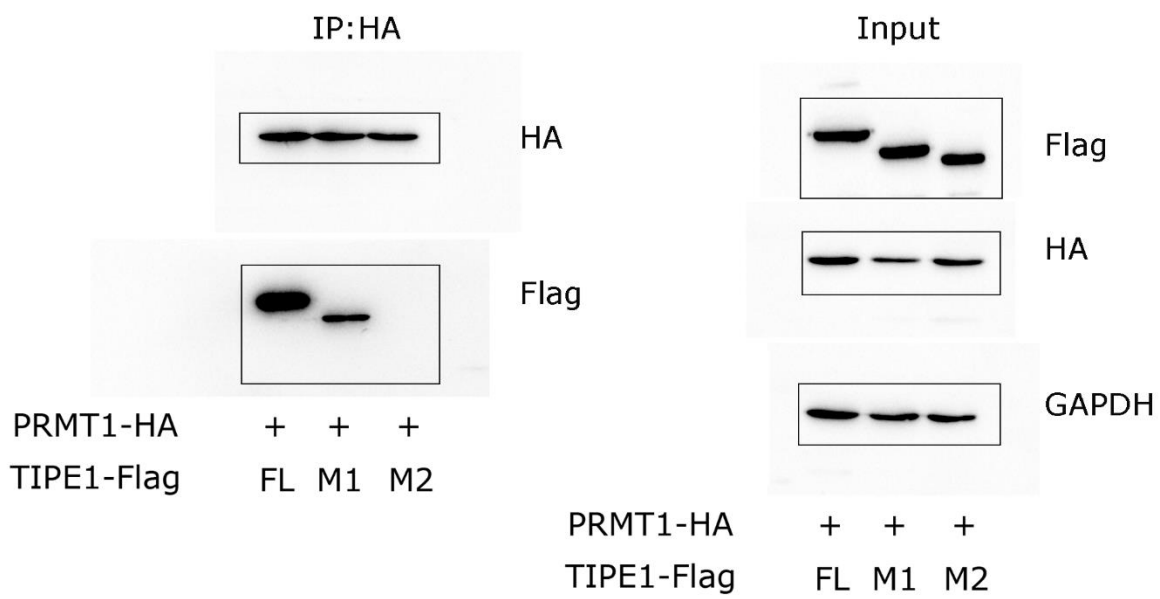

**Fig. 6G**

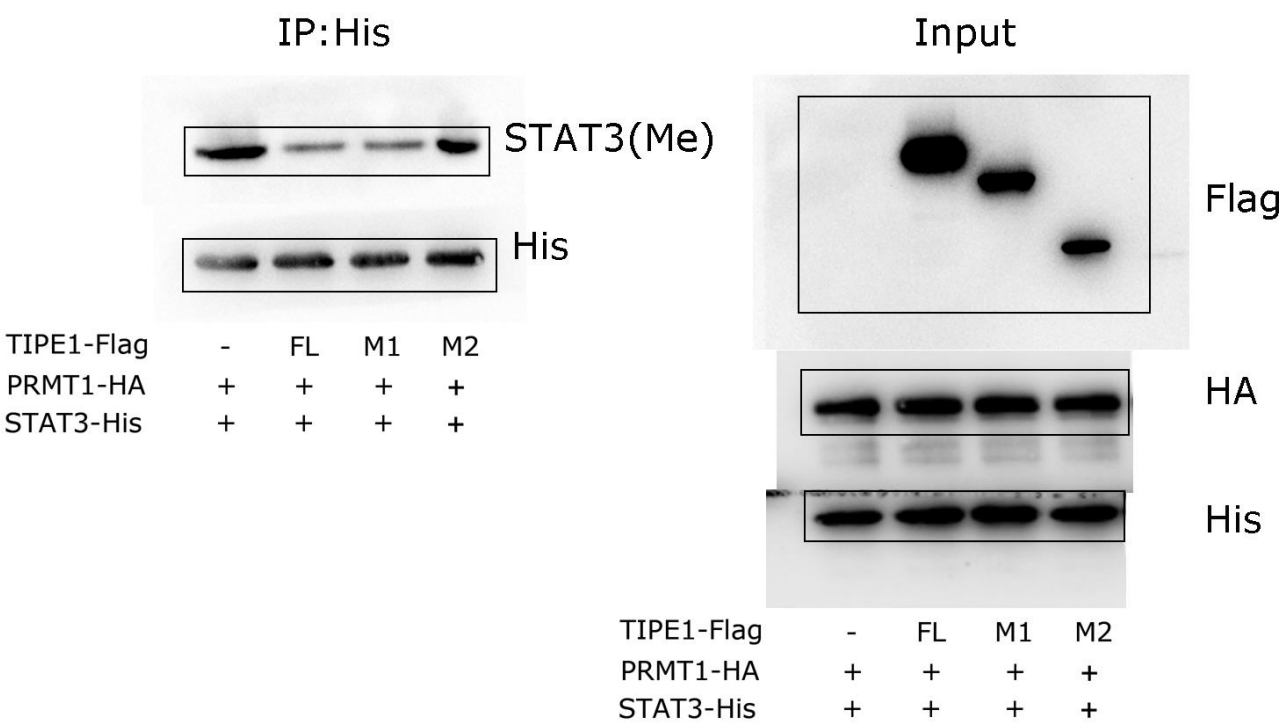

**Fig. 7B**

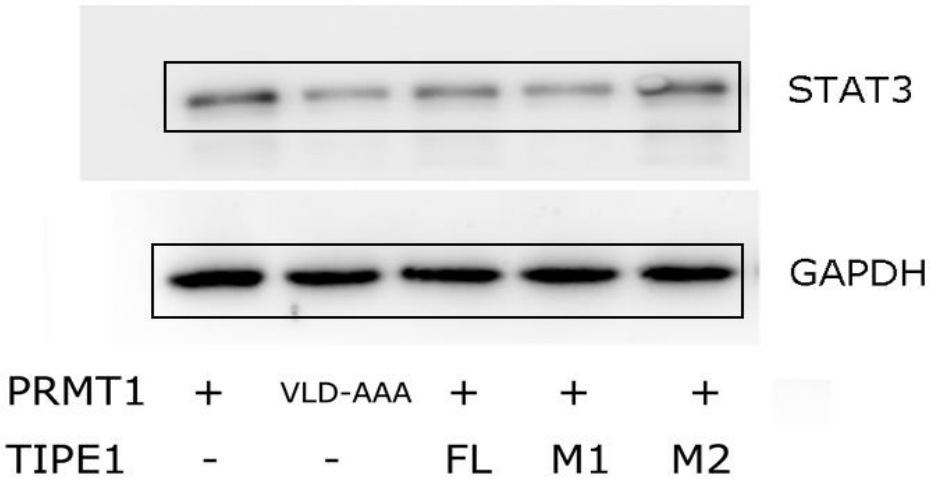

**sFig. 1**

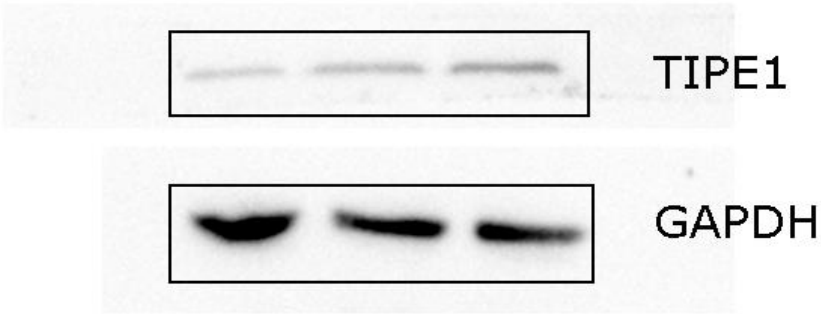

**sFig.3**

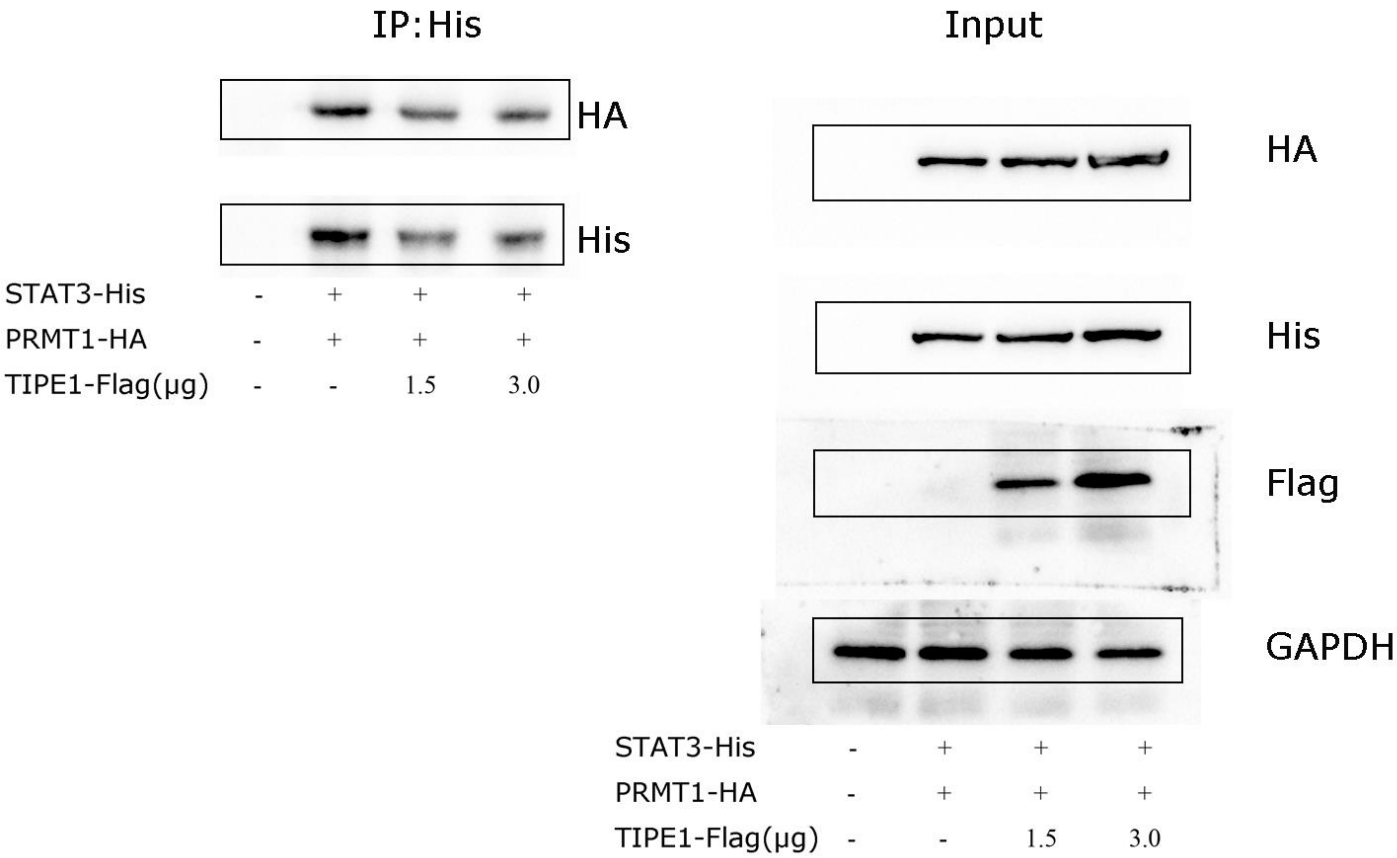

Supplement: Supplementary file 3 — Full Western Blots [file 41419_2022_5273_MOESM3_ESM.pdf]
